# Leaf development: a cellular perspective

**DOI:** 10.3389/fpls.2014.00362

**Published:** 2014-07-31

**Authors:** Shweta Kalve, Dirk De Vos, Gerrit T. S. Beemster

**Affiliations:** ^1^Laboratory for Molecular Plant Physiology and Biotechnology, Department of Biology, University of AntwerpAntwerp, Belgium; ^2^Department of Mathematics and Computer Science, University of AntwerpAntwerp, Belgium

**Keywords:** leaf growth, developmental pathway, plant hormones, stress, modeling

## Abstract

Through its photosynthetic capacity the leaf provides the basis for growth of the whole plant. In order to improve crops for higher productivity and resistance for future climate scenarios, it is important to obtain a mechanistic understanding of leaf growth and development and the effect of genetic and environmental factors on the process. Cells are both the basic building blocks of the leaf and the regulatory units that integrate genetic and environmental information into the developmental program. Therefore, to fundamentally understand leaf development, one needs to be able to reconstruct the developmental pathway of individual cells (and their progeny) from the stem cell niche to their final position in the mature leaf. To build the basis for such understanding, we review current knowledge on the spatial and temporal regulation mechanisms operating on cells, contributing to the formation of a leaf. We focus on the molecular networks that control exit from stem cell fate, leaf initiation, polarity, cytoplasmic growth, cell division, endoreduplication, transition between division and expansion, expansion and differentiation and their regulation by intercellular signaling molecules, including plant hormones, sugars, peptides, proteins, and microRNAs. We discuss to what extent the knowledge available in the literature is suitable to be applied in systems biology approaches to model the process of leaf growth, in order to better understand and predict leaf growth starting with the model species *Arabidopsis thaliana*.

## INTRODUCTION

Understanding the regulation of plant growth and its constituent organs is an important objective in biology. It forms the basis for crop yield, turn-over in ecosystems and the means for the plant to adapt to environmental conditions and experimental treatments. The development of leaves in dicotyledonous plant species is an intriguing process, resulting from a complex interplay of a multitude of regulatory pathways. On the one hand it is so strictly regulated that the resultant leaf morphology is a reliable characteristic for taxonomic classification. On the other hand however, the process is so plastic that environmental factors can affect mature leaf size by an order of magnitude. Curiously, leaf shape is often largely conserved between related species with genetic variations in thousands of genes, while a single mutation can sometimes induce morphological differences similar to those that distinguish species and even families (e.g., Barkoulas et al., 2008). Due to these intriguing characteristics and the importance of leaves for plant performance and function, many aspects of leaf development have been extensively studied.

In recent decades, remarkable progress has been made in understanding the regulation of leaf development *via* molecular/genetic approaches. Moreover, increasing use of high-throughput technologies is constantly providing new biological information at various organizational levels. In this context, systems biology provides a means to integrate the accumulating knowledge into holistic mechanistic models to get a complete understanding of biological processes. These models are often implemented through computer simulations of normal and/or experimentally perturbed systems to test how well they resemble the real situation and increase our understanding of its mechanistic basis.

**FIGURE 1:**
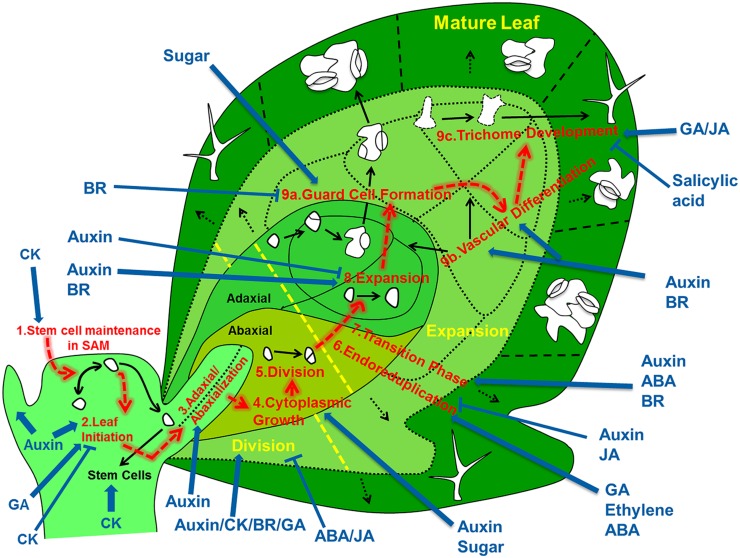
**Overview of the regulatory processes that determine the development of a leaf.** The cells that form the leaf originate from the stem cell niche at the shoot apical meristem. As a first step in their development, cells need to loose stem cell identity (1). A leaf primordium is initiated in groups of cells that migrate into the lateral regions of the SAM (2), which further acquires upper (adaxial) and lower (abaxial) sides through leaf-polarity control (3). Afterward, the transformation of the small leaf primordium to a mature leaf is controlled by at least six distinct processes: cytoplasmic growth (4), cell division (5), endoreduplication (6), transition between division and expansion (7), cell expansion (8) and cell differentiation (9) into stomata (9a), vascular tissue (9b), and trichomes (9c). Most of these processes are tightly controlled by different signaling molecules, including phytohormones. The developmental path of cells is indicated with red arrows, key regulatory processes are numbered and indicated and regulation of these processes by phytohormones/sugar is shown by blue arrows (pointed and T shaped arrows indicate positive and negative regulation, respectively).

A mechanistic understanding of leaf development should encompass an integrated view on the regulatory networks that control developmental decisions and processes of cells as they migrate in space and time from the shoot apical meristem (SAM) to their final position in the leaf (**Figure 1**). Therefore, we review the subsequently acting developmental networks that guide individual cells on their way from the SAM to their differentiated state somewhere in a fully differentiated leaf. Based on this description we delineate to what extent we understand how variations in the regulation at the cell level affect the shape and size of the leaf as a whole, and what are the implications for implementing this knowledge into fully fledged simulation models.

## PROCESSES THAT CONTROL LEAF GROWTH

The development of a leaf is a dynamic process where independent regulatory pathways instruct component cells at different stages of their development to make differentiation switches and to regulate the rate at which developmental processes are executed. Each of these regulatory control points is essential to steer the development of individual cells. When integrated over the entire cell population of a leaf, its growth and ultimately size and shape are emergent properties that can be compared to real leaves. Because developmental signals are perceived and executed at the level of individual cells, it is essential to understand how these signals are integrated in the leaf developmental process, which can be achieved by modeling the path of an individual cell (and its progeny) from SAM to the mature leaf. Although many of the pathways involved have been extensively reviewed, to our mind the perspective of the individual cells has not been explored systematically. Therefore the main aim of the present review is to provide this cellular perspective to leaf development.

### THE SHOOT APICAL MERISTEM

The SAM is the source of all cells that ultimately form the shoot, including the subset that ends up building the leaves. Generally, cells in the central zone (CZ) of the SAM divide at a relatively low rate and remain in an undifferentiated state, whereas cells at the peripheral zone (PZ) divide faster and differentiate into organs such as leaves, axillary nodes, and floral parts (Veit, 2004; Braybrook and Kuhlemeier, 2010). In dicots, the SAM consists of three layers L1, L2, and L3; epidermal (L1) and subepidermal (L2) layers are known as tunica and the inner layer (L3) is called the corpus (Satina et al., 1940).

**FIGURE 2:**
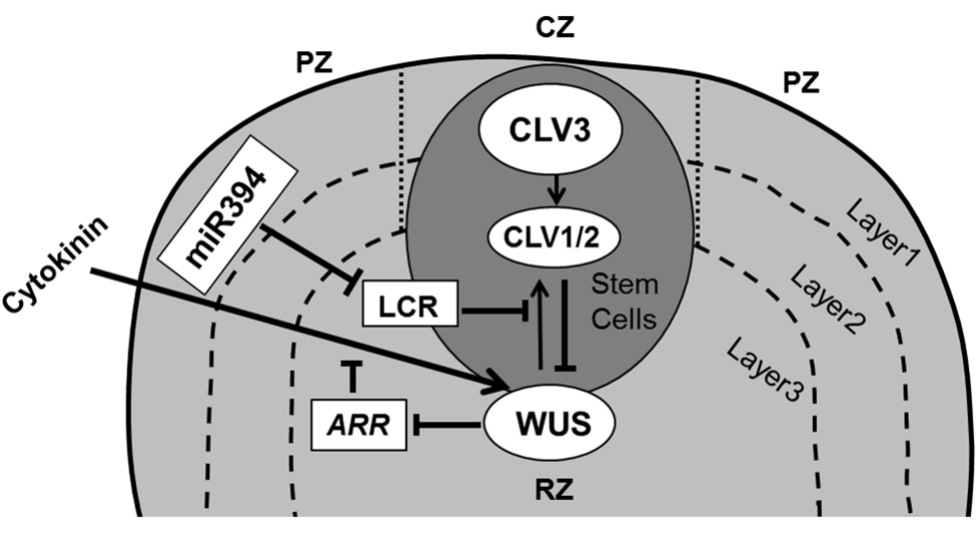
**Maintenance of stem cells in shoot apical meristem.** The SAM is organized in three functional zones [central zone (CZ), peripheral zone (PZ), and rib zone (RZ)] and three layers where the antagonistic relation between WUS and CLV is essential to preserve cells in the meristem. WUS activates CLV3, which further binds with CLV1/2 and in turn inhibits expression of *WUS*. Cytokinin positively controls *WUS* expression where *ARRs* are negative regulators of cytokinin and are inhibited by WUS. The L1 specific miR394 negatively affects the LCR protein, which interferes in *WUS/CLV* based stem cell maintenance (pointed and T shaped arrows indicate positive and negative regulation, respectively).

From the cellular perspective, on-going (slow) division in the stem cell niche will cause cells to become displaced away from the quiescent center, where at some well-defined place they lose their stem cell fate and acquire the actively dividing state. This transition is controlled by the interplay of a regulatory loop involving the homeodomain transcription factor WUSCHEL (WUS) in the rib zone (RZ) and *CLAVATA* gene products (CLV1, CLV2, and CLV3) expressed in the CZ of the SAM (Brand et al., 2000; Schoof et al., 2000; Carles and Fletcher, 2003; Yadav and Reddy, 2011). The WUS and CLV based pathway operates through two mobile signals: CLV3 and a hypothetical WUS mediated signal (**Figure 2**). *CLV3* encodes a small secreted ligand that is produced specifically in L1 and L2 cells, and moves into the underlying L3 cells where it binds with receptor like proteins CLV1 (LRR receptor kinase) and/or CLV2 (receptor-like protein), which in turn inhibit WUS activity (Clark, 2001; Carles and Fletcher, 2003). WUS activity in the L3 cells induces the production of a non-cell-autonomous signal that moves to the stem cells and activates the expression of *CLV3* there (Haecker and Laux, 2001; Braybrook and Kuhlemeier, 2010). It was proposed that the L1 produced miR394 signal is necessary for spatial organization of the SAM. This mobile microRNA regulates WUS mediated stem cell maintenance by inhibition of F box protein LEAF CURLING RESPONSIVENESS (LCR; Knauer et al., 2013).

Upon mutation in *WUS* the stem cells precociously transit into the peripheral actively dividing zone, ultimately consuming the stem cell niche and thereby the meristem. Inversely, in *clv1* and *clv3* mutants WUS activity of SAM cells is maintained much longer, whereby the stem cell niche and consequently the SAM as a whole enlarges dramatically (Clark et al., 1993, 1995; Laux et al., 1996). Several mathematical models have focused on the WUS–CLV interaction, predicting to various degrees how their expression domains are modulated through mutation or misexpression (Jonsson et al., 2005; Nikolaev et al., 2007; Hohm et al., 2010). Recent experimental studies supported by mathematical modeling have shown that WUS movement is essential for direct transcriptional repression of the differentiation program (Yadav et al., 2013) as well as in restricting its own accumulation through activating its negative regulator CLV3 (Yadav et al., 2011).

It has been postulated that signaling by the plant hormone cytokinin (CK) regulates *WUS* expression via CLV-dependent and CLV-independent mechanisms (Gordon et al., 2009) to promote SAM growth and maintenance with WUS repressing the transcription of *ARABIDOPSIS* type-A* RESPONSE REGULATORS (ARRs)*, which are the negative regulators of CK signaling (Leibfried et al., 2005; Sablowski, 2007; **Figure 2**). Indeed, mutations in CK receptors (Higuchi et al., 2004) and over-expression of the CK dehydrogenase gene family of *Arabidopsis* (AtCKX; Werner et al., 2003) reduce meristem size and leaf area, indicating a relation between the SAM and leaf size. It appears however that that the number of leaf founder cells is not an important determinant of the final leaf size. For instance, a meta-analysis across a wide range of cactus species indicates that the size of the SAM correlates closely to the number of leaves formed and has only minor implications for their ultimate size (Mauseth, 2004).

### LEAF INITIATION

**FIGURE 3:**
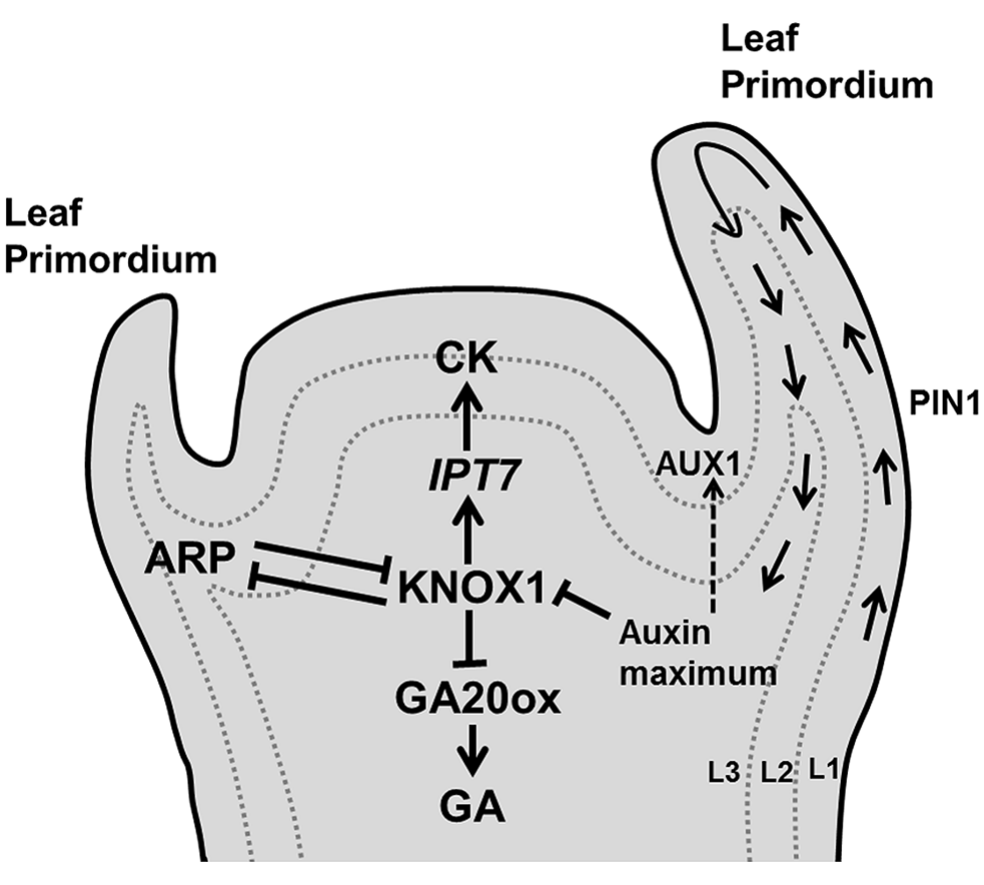
**Decision of leaf initiation.** Accumulation peaks of auxin at the flank of the SAM through PIN1/AUX1 mediate polar auxin transport, triggers development of a primordium where KNOX1 plays key role in stem cell maintenance. Additionally, KNOX1 positively regulates CK whereas it negatively affects GA signaling through *IPT7* and GA20 oxidases, respectively. Opposite to it, ARP regulates the emergence of a young primordium (pointed and T shaped arrows indicate positive and negative regulation, respectively).

Once progenitor cells are outside the stem cell niche, they need to decide whether they will contribute to the main axis or will differentiate into lateral appendices such as leaf primordia. This decision is primarily governed by the accumulation of the plant hormone auxin its influx carrier [AUXIN RESISTANT (AUX1) and its PIN-FORMED1 (PIN1)] eﬄux transporter (Bayer et al., 2009; Guenot et al., 2012). The eﬄux carriers orient the transport of auxin toward neighboring cells with a higher auxin concentration, leading to the formation of accumulation patterns across the cell population. Several mathematical modeling studies (reviewed in De Vos et al., 2012) have simulated phyllotactic patterning based on feedback interactions between auxin and PIN distribution. Some models postulate that AUX1 creates auxin accumulation mainly in L1 layer cells, whereas PIN1 is initially localized in the protodermal (L1) layer cells and causes drainage of auxin toward the base of the shoot by inducing vascular strand differentiation in L2/3 layer cells of the SAM (Reinhardt et al., 2003; de Reuille et al., 2006; **Figure 3**).

*Arabidopsis* leaf differentiation from the apical meristem is abolished in the auxin biosynthetic triple mutant *yuc1 yuc4 pin1* (Cheng et al., 2007), whereas longer plastochron and irregular vegetative growth occurs in the *pin1* mutant (Guenot et al., 2012). Cells in the SAM, which serve as a stem cell population, differ from the cells in the leaf primordium. This distinction is controlled by complex and still poorly characterized regulatory networks in which the antagonistic relation between two families of transcription factors, KNOTTED-like homeobox (KNOX1) and ASYMMETRIC LEAF1/ROUGH SHEATH2/PHANTASTICA (ARP) proteins (Byrne et al., 2002; Hay and Tsiantis, 2006, 2010) plays a crucial role (**Figure 3**). *KNOX* is expressed in all meristem cells except those at the site of the organ initiation (Jackson et al., 1994; Long et al., 1996), whereas the (*ARP* family) *AS1* mRNA is expressed in the primordia forming cells, but not in the meristem (Byrne et al., 2000). *KNOX1* is required to maintain undifferentiated cells in the SAM (Scofield and Murray, 2006) and increases CK biosynthesis (Yanai et al., 2005), whereas *ARP* initiates differentiation in the leaf primordium (Byrne et al., 2002). High levels of auxin restrain CK biosynthesis by the repression of KNOX1 activity (Su et al., 2011; **Figure 3**). High auxin and AS1 expression also suppress the expression of the KNOX gene *BREVIPEDICELLUS (BP)*, which is required for leaf initiation (Hay et al., 2006). In *Arabidopsis*, the *KNOX1* gene* SHOOTMERISTEMLESS (STM)* acts antagonistically with *ARP* gene products to control the induction of leaf primordia. The loss of function mutant *stm* fails to produce a SAM, also preventing leaf formation (Long et al., 1996), whereas the *as1* and *as2* mutants have small and round leaves (Byrne et al., 2002).

It has been demonstrated that the KNOX proteins trigger CK biosynthesis through the activation of *IPT7* (encodes the CK biosynthetic enzyme isopentenyl transferase) and repress the transcription of gibberellin (GA) biosynthetic genes that encode GA20-oxidases (Sakamoto et al., 2001; Jasinski et al., 2005; **Figure 3**). Thus, high CK and low GA maintain stem cell identity in SAM cells by preventing cell differentiation (Gordon et al., 2009; Veit, 2009).

### LEAF POLARITY

After acquiring “leaf” identity, the cells in the primordium have to develop a polarity gradient along the dorso-ventral axis. Once the position of the leaf primordium is established, a further increase in cell proliferation rates stimulates primordium outgrowth from the SAM. If this growing primordium is removed by tangential incision, another primordium arises which is cylindrical and abaxialized (lacking a flat leaf blade). This highlights the importance of signals originating in the SAM and received by cells in the primordium to determine polarity. This so called Sussex signal is yet to be identified (Sussex, 1951). Waites and Hudson (1995) proposed that the dorsal and ventral sides of the leaf are specified in the early development of the leaf primordium, when it is still located within the SAM. They showed that the *PHAN* gene (encoding a MYB type transcription factor) in *Antirrhinum majus* is involved in ab/ad-axial leaf polarity. Subsequently the *phabulosa-1 (phb-1d)* mutant was characterized in *Arabidopsis* whose leaves were unable to develop a blade and were radially symmetrical (McConnell and Barton, 1998).

**FIGURE 4:**
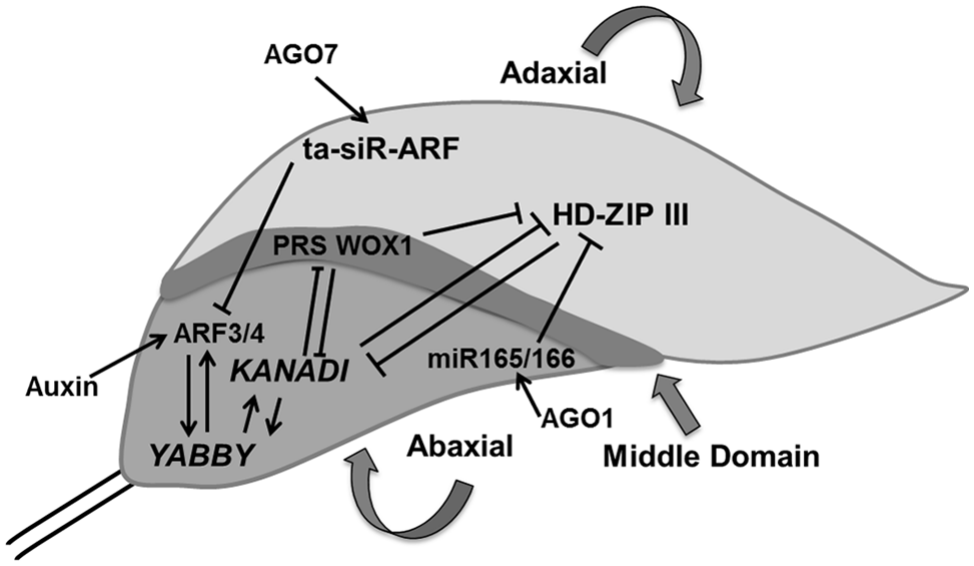
**Polarity control.** The young leaf primordium has three domains which are determined by domain specific transcription factors such as HD-ZIP III, KANADI, and PRS WOX1 for adaxial, abaxial, and middle regions, respectively. These transcription factors inhibit expression of each other and thereby control their expression in another domain. AGO1 regulates miR165/66 which inhibits HD-ZIP III whereas AGO7 stabilizes ta-siR-ARF which causes the degradation of ARF3/4, which itself is controlled by auxin. *YABBY* determines the abaxial side in cross talk with KANADI (pointed and T shaped arrows indicate positive and negative regulation, respectively).

Our knowledge of the regulation of antagonistic transcription factors specifying upper and lower sides has greatly increased, but the molecular signals exchanged between cells on both sides of the primordium to create this polarity are yet to be identified. Adaxial domain identity is determined by the expression of *PHABULOSA* (*PHB*),* PHAVOLUTA* (*PHV*), and *REVOLUTA* (*REV*) genes, which encode class III homeodomain-leucine zipper (HD-ZIPIII) proteins (McConnell et al., 2001). The identity of cells in the abaxial domain depends on the expression of* KANADI* [*KAN*; which encodes a Golden2/*Arabidopsis* response-regulator/P starvation/acclimatization response (Psr1; GARP) transcription factor; Eshed et al., 2001; Kerstetter et al., 2001] and the *YABBY* gene family (Siegfried et al., 1999; Eshed et al., 2004). These two classes of genes produce signals that suppress each other’s expression: the expression of *PHB/PHV/REV* genes in cells located at the abaxial side is inhibited de by *KAN* and inversely *KAN* expression in abaxially located cells in inhibited by the activity of *PHB/PHV/REV* genes, providing a feedback communication between the two sides (Tsukaya, 2013b; **Figure 4**).

Two small RNAs, the 21-nucleotide microRNA (miR165/166) and the 24-nucleotide transacting small interfering RNA (ta-siRNA), ta-siR-ARF, are also involved in determining leaf polarity (Chitwood et al., 2007, 2009; Nogueira et al., 2007). *ARGONAUTE1* (*AGO1*) affects the regulation of miR165/166, which stimulates the cleavage of *HD-ZIPIIIs* transcripts in cells located on the adaxial side (Kidner and Martienssen, 2004) whereas, *AGO7/ZIPPY* (*ZIP*) stabilizes ta-siR-ARF, which further targets the degradation of auxin-related transcription factors, *ETTIN (ETT)/ARF3* and *ARF4* on the abaxial side (Adenot et al., 2006; Hunter et al., 2006). *FILAMENTOUS FLOWER/YABBY3 (FIL/YAB3)*, a member of the *YABBY* family, up-regulates *KAN1* and *ARF4*, which establishes a positive feedback loop (Bonaccorso et al., 2012; **Figure 4**).

Differences in cell growth rates along the principal developmental axes are crucial in determining final leaf shape. In addition to specification of adaxial and abaxial side of the leaf, margin specific cell fate is induced in cells residing at the boundary between these two surfaces (McHale, 1993). In contrast to regulation of leaf blade outgrowth, the influence of ad/abaxial specific genes on marginal cells is yet to be explored. Recently, middle leaf domain specific *WUS-RELATED HOMEOBOX* (*WOX*) genes were reported, which affect leaf blade outgrowth and margin specific development (**Figure 4**). These transcription factors (WOX1 and PRESSED FLOWER (PRS), i.e., WOX3) are repressed by KAN. The loss of function of *WOX1* and *PRS* causes instable organization of ad-abaxial polarity (Nakata and Okada, 2012; Nakata et al., 2012).

A relatively simple computational model supported by time-lapse data and clonal analysis by Kuchen et al. (2012) accounts for local differences in cell growth rates and direction driving organ level shape changes during early *Arabidopsis* leaf development. A central model assumption is a yet uncharacterized early tissue polarity system that deforms during growth. *CUP-SHAPED COTYLEDON* (*CUC*) genes are emerging as prime candidate organizers of tissue polarity (Hasson et al., 2011). Correspondence of cell polarity and PIN1 auxin transporter patterns points to their involvement in such an organizer-based model (Scarpella et al., 2006). Moreover, it was reported that the outgrowth of lobes at the leaf margin is specified by a local auxin maximum as a result of the polar distribution of the PIN1 transporter (Hay et al., 2006). The transcription factor CUC2 which is expressed at the leaf sinuses and is negatively regulated by *miR164* (Nikovics et al., 2006), promotes generation of these PIN1-dependent auxin maxima, which was supported by computer simulations (Bilsborough et al., 2011). Recently, a homeodomain protein REDUCED COMPEXITY (RCO) was reported to enhance serration by repressing growth at the flanks of initiating leaves (Vlad et al., 2014). Interestingly, CUC2, PIN1, and TEOSINTE BRANCHED/CYCLOIDEA/PCF (TCP), and KNOX have been implicated in compound leaf development illustrating that this regulatory system has the capacity to take more extreme forms than observed in *Arabidopsis* leaves (Barkoulas et al., 2008; Hay and Tsiantis, 2010; Koyama et al., 2010). A better understanding of the regulatory mechanisms operating at the cell level during the early phases of leaf outgrowth will likely provide invaluable insights into how diverse leaf morphologies are established.

### CYTOPLASMIC GROWTH

In contrast to the morphology of the leaf primordium, the final size and shape of the leaf differ widely among species. Differences in leaf outgrowth are often interpreted as the result of cell division producing a certain number of cells and subsequent cell expansion determining their mature size. However, this is an overly simplistic view. Firstly, the relationship between cell division and expansion is complex and the two processes can mutually compensate each other (Tsukaya, 2002; Beemster et al., 2003). A theoretical framework to understand this phenomenon was provided by Green (1976), who proposed that cell growth and partitioning (division *sensu-strictu*) are two processes that co-occur in proliferating cells, whereas in expanding cells cell-growth continues in absence of partitioning. Clearly, this framework allows for continued growth irrespective of inhibited or stimulated cell division activity, at least until cells get too small or too large to function normally. However, the view that cell growth in proliferating cells is equivalent to that in expanding cells is overly simplistic. It is clear that whereas dividing cells grow by increasing cytoplasmic volume, expanding cells primarily increase their internal volume by expanding their vacuolar volume.

**FIGURE 5:**
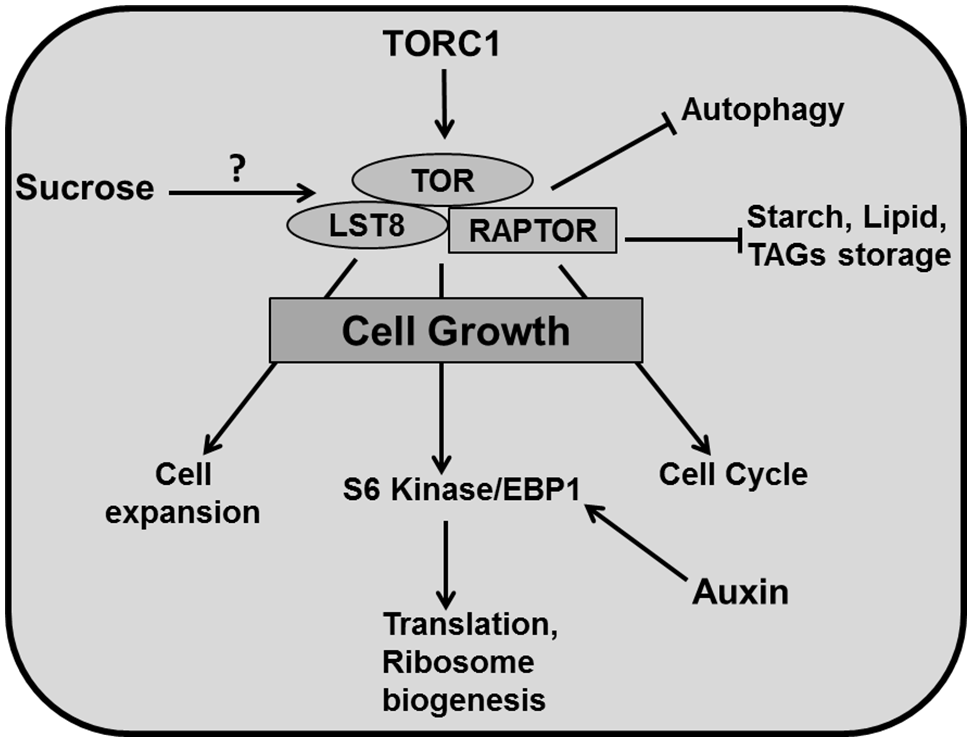
**Cytoplasmic growth.** TOR is the central regulator of diverse growth processes. TOR, RAPTOR, and LST8 are major components of TORC1 in plants. TOR has been reported to regulate different metabolic processes and positively controls cell expansion, cell cycle, translation, ribosome biogenesis (through phosphorylation of S6 kinase/EBP1). TOR activity inhibits autophagy and accumulation of carbon resources such as starch and lipids like triacylglycerides (TAGs). Auxin positively regulates EBP1 proteins. It has been reported that sucrose positively affects TOR activity (pointed and T shaped arrows indicate positive and negative regulation and question mark indicates an unknown mechanism, respectively).

Cytoplasmic growth is mainly based on macromolecular synthesis and therefore consumes a lot of energy. A crucial role in ensuring a sufficient supply of elementary building blocks is played by the Target of Rapamycin (TOR) pathway. TOR, a Serine/Threonine kinase of the phosphatidylinositol-3-kinase-related kinase (PIKK) family is an essential controller of cytoplasmic growth and metabolism in plant cells. It controls a range of cellular responses such as ribosome biogenesis, translational initiation, cell proliferation, cell expansion and autophagy (Zhang et al., 2013; Sablowski and Dornelas, 2014; **Figure 5**).

In yeast and animals, there are two TOR complexes: TORC1 and TORC2 whereas in plants there is only evidence for TORC1. TOR, Regulatory associated protein of TOR (RAPTOR), and Lethal with Sec13 protein 8 (LST8) are three key components of TORC1 in *Arabidopsis* (Moreau et al., 2012). In contrast to other eukaryotes, much less is known about TOR signaling in plants. *Arabidopsis thaliana* is insensitive to the drug rapamycin, which is extensively used to study TOR function in yeast and animal systems, which has formed a major obstacle to study TOR in plants (Menand et al., 2002; Anderson et al., 2005; Deprost et al., 2007; Sormani et al., 2007). However, a recent study shows that plants do respond to rapamycin at the concentration of 10 μM, which is 100 times more than the concentration used for yeast and mammals (Xiong and Sheen, 2012). Mutation in *TOR* is embryo-lethal in plants (Menand et al., 2002; Ren et al., 2011) and therefore, an alternative approach of inducible knockout for conditional inhibition of *TOR* has to be used for functional studies (Deprost et al., 2007; Caldana et al., 2013).

An* Arabidopsis* T-DNA insertion line which overexpresses *AtTOR* produces bigger leaves containing larger cells (Deprost et al., 2007). Accordingly, down-regulation of *TOR* by inducible artificial microRNA produced smaller leaves with fewer cells along with up-regulation of metabolic pathways like the Krebs cycle. These lines also accumulated storage products such as starch and lipids like triacylglycerides (TAGs) displaying reduced growth (Caldana et al., 2013). Mutation of *LST8*, a member of the TOR complex (TORC1) decreased plant size by producing fewer and smaller leaves alongside a higher amount of starch and amino acids and reduced sucrose concentrations. Moreover, down-regulation of genes involved in cell wall formation like expansins (EXPs) and *CELLULOSE SYNTHASE-LIKE G3* in this mutant demonstrates a role for TOR in cell expansion (Moreau et al., 2012). The TORC1 component RAPTOR accumulates in dividing and expanding cells whereas mutation in *AtRaptor1B* slows down the leaf initiation. A double mutant of *AtRaptor1A* and *AtRaptor1B* exhibited normal seedling growth, but was unable to maintain post embryonic development (Anderson et al., 2005).

TORC1 promotes the phosphorylation of S6 kinase (S6K) and eIF4E-binding proteins (E-BP1), which controls translation and ribosome biogenesis (Krizek, 2009). Again, auxin is involved through the regulation of EBP1 protein stability in *Arabidopsis* (Horvath et al., 2006; **Figure 5**). TORC1 also regulates autophagocytosis which ensures synthesis, degradation and recycling of cellular components (Sablowski and Dornelas, 2014). Recent studies have revealed that the TOR pathway is an essential controller for cellular development, which regulates cell expansion and cell cycle simultaneously. The TOR pathway is directly connected to cell cycle regulation by mediating E2Fa phosphorylation and activation of DNA synthesis genes (Xiong et al., 2013) and regulates cell wall modification and degradation processes like senescence and autophagy (Caldana et al., 2013). Importantly, there is a link between the TOR pathway and nutrient status. TOR plays a central role in connecting growth related genes to glucose signaling (Xiong et al., 2013).

Given this central role in connecting growth regulation to nutritional status, the TOR pathway is a key regulatory hub in organ development. Although molecular insight of its functioning in animal systems is rapidly increasing, currently we are not aware of mechanistic models that include this knowledge, a void that we expect to be filled in the coming years.

### CELL DIVISION

**FIGURE 6:**
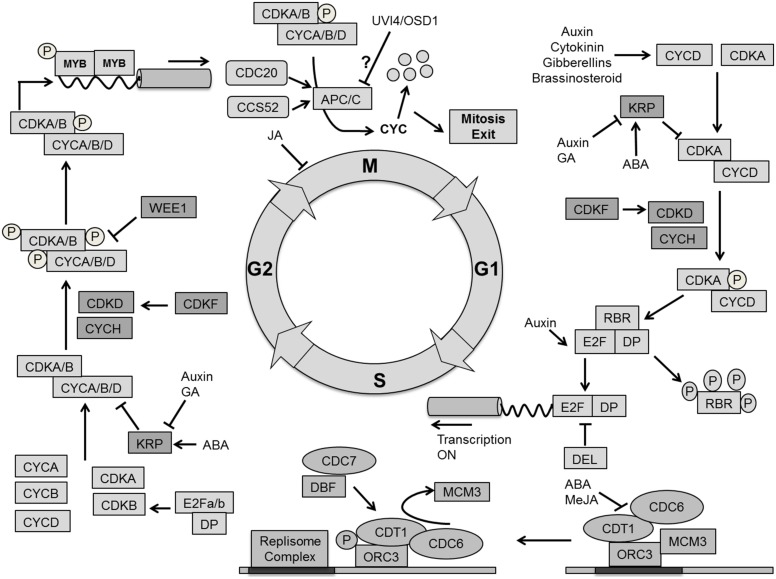
**Molecular mechanism for cell cycle regulation.** Four phases of cell cycle (G1, S, G2, and M) are operated by successive activation and deactivation of cyclin dependent kinases (CDKs). During the cell cycle these kinases bind with cyclins and get activated through phosphorylation by CDK activating kinases (CDKD and CDKF) whereas KRPs inhibit the complexes. G1 to S transition is controlled by CDKA–CYCD which phosphorylates the RBR proteins and releases the E2F transcription factor, which activates S phase related genes. The G2–M transition is dependent on CDKA/B and CYCA/B/D. The CDK complex is inactivated by phosphorylation through WEE1. The exit from mitosis requires proteolytic degradation of CYCs which as mediated by the Anaphase-Promoting Complex/Cyclosome (APC/C) bind with CCS52 and CDC20. Phytohormones like auxin, cytokinin, gibberellins (GA), brassinosteroids, abscisic acid (ABA) and methyl jasmonate (MeJA) impact cell cycle regulation at different points (pointed and T shaped arrows indicate positive and negative regulation and question mark indicates unknown regulation, respectively).

In addition to cell volume increase by cytoplasmic growth, cell proliferation exponentially increases the number of cells in the developing leaf. In general cell growth needs to be sufficiently balanced by cell division for stable tissue growth (Sablowski and Dornelas, 2014). The cell division cycle is a unidirectional process, tightly regulated by a molecular mechanism that is largely conserved between all eukaryotes (Inze et al., 1999; Dewitte and Murray, 2003; Inze and De Veylder, 2006; **Figure 6**).

The plant cell cycle is controlled by the activity of complexes consisting of a cyclin-dependent kinase as the catalytic subunit and a cyclin as the regulatory subunit. A-type cyclin dependent kinase (CDKA) and D-type cyclin (CYCD) are central to the G1/S phase transition in which the cell activates DNA duplication. CDKA is a key protein to control cell division in *A. thaliana*, and is present at a constant level throughout the cell cycle (Porceddu et al., 2001; Joubes et al., 2004; Gaamouche et al., 2010). Overexpression of a dominant negative *CDKA;1* of *A. thaliana* in tobacco plants inhibited cell division rate, resulting in the formation of fewer, but larger cells resulting in an overall reduction of leaf area (Hemerly et al., 1995). It has also been demonstrated that the *CDKA;1* activity maintains the SAM cells in an undifferentiated state. Expressing a dominant negative allele of CDKA;1 in the *STM* domain of the shoot apex produces smaller leaves with a reduced number of epidermal cells (Gaamouche et al., 2010). Interestingly, irregularly shaped epidermal cells observed in a *CDKA;1* dominant negative mutant expressed from the *STM* promoter point toward *CDKA* influencing cell wall and cytoskeleton properties (Borowska-Wykret et al., 2013). A triple *cycd3;1–3* loss of function mutant in the *Arabidopsis* leaf shows a decreased cell number and reduced CK response (Dewitte et al., 2007). The plant hormones auxin, CK, brassinosteroid (BR), and GAs increase the level of CYCD, thereby activating CDKA (Riou-Khamlichi et al., 1999; Francis and Sorrell, 2001; Inze and De Veylder, 2006; Perrot-Rechenmann, 2010). The repression of ABP1 (AUXIN BINDING PROTEIN1) negatively influences transcript levels of CYCDs, which results in impaired cell division in the leaf (Braun et al., 2008), whereas BRs up-regulate the expression of CYCD3 and promote cell division through a mechanism that requires *de novo* protein synthesis (Hu et al., 2000). The BR biosynthesis mutant *det2* (*de-etiolated2* = *cro1*) and *dwf1* (*dwarf1* = *cro2*) produce fewer cells and a smaller leaf, which can be reversed by brassinolide application, indicating a dual role of BR in division and expansion (Nakaya et al., 2002).

The activity of CDKA/CYCD complexes is itself controlled by CDK activating kinases CDKD and CDKF coupled with CYCH, which activates the complex through a phosphorylation cascade. CDKF;1 was also found to activate CDKD;2 and CDKD;3 by T-loop phosphorylation (Umeda et al., 2005; Takatsuka et al., 2009). The active CDKA/CYCD complex triggers the dissociation of E2F/DP heterodimeric complex from RBR (retinoblastoma-related protein) through phosphorylation. Additionally, it initiates the destruction of E2Fc/DP/RBR transcriptional repressor complex by the Skp-Cullin1-F-Box (SCF) E3-ubiquitin protein ligase (Inze and De Veylder, 2006). The RBR protein regulates the activity of E2F transcription factors to control cell proliferation. It is an essential regulator of the cell cycle to coordinate between cell division, differentiation and homeostasis (Desvoyes et al., 2006; Borghi et al., 2010). Once the E2F/DP complex is separated from RBR, it initiates G1 to S transition by activating the transcription of genes required for DNA duplication. Furthermore, the E2F-like DEL transcription factors compete with E2F/DP for DNA binding sites (**Figure 6**).

*Arabidopsis* has three typical E2Fs (E2Fa, E2Fb, and E2Fc), two dimerization proteins (DPa and DPb) and three atypical E2Fs (E2F/DEL2, E2FE/DEL1, and E2Ff/DEL3; Mariconti et al., 2002). E2Fa and E2Fb stimulate entry into and progression of the S-phase and overexpression of these transcription factors leads to an enlarged phenotype due to enhanced cell proliferation (De Veylder et al., 2002; Sozzani et al., 2006). Auxin positively regulates E2Fb protein levels (Magyar et al., 2005). Additionally, the *AXR1* transcript level was found to be high in an E2Fb overexpression line (Sozzani et al., 2006). On the other hand, E2Fc is a negative regulator of the S-phase where a decreased level leads to leaves and cotyledons with more but smaller cells (del Pozo et al., 2006). Auxin affects the E2Fc protein level in *Arabidopsis*. Mutation in the *AXR1* gene leads to impaired modification of the CUL1 protein, a structural component of the E3–SCF complex, with the Ub-related protein RUB, and shows increased E2Fc protein levels (del Pozo et al., 2002). Atypical E2Fs/DELs are transcriptional regulators for endoploidization, which act independently of DPs and RBR. It is still unclear if they compete with typical E2Fs for binding sites or actively repress gene transcription (Vlieghe et al., 2005; Berckmans and De Veylder, 2009).

Activated E2Fs in *Arabidopsis* target the expression of genes involved in DNA repair and chromatin dynamics such as CDC6, CDT1, MCM3, ORC1 and ORC3, RNR, and PCNA. However, they also influence the expression of genes responsible for G2-M transition like CDKB1, MYB, and ANAPHASE-PROMOTING COMPLEX/CYCLOSOME (APC/C; de Jager et al., 2001; Ramirez-Parra et al., 2003; Boudolf et al., 2004b; Vandepoele et al., 2005; Lammens et al., 2008; Naouar et al., 2009). Replication origin sites are silenced in the G1 phase where CDC6 and CDT1 together with ORC allow the loading of MCM to the replication origins; hence promoting the complex for activation of the S phase. Later, the DBF–CDC7 complex phosphorylates ORC which then moves and exposes the replication initiation site for the replisome complex, allowing replication to start (Blow and Dutta, 2005; Francis, 2007). The plant hormone ABA negatively regulates the expression of the *CDT1a* gene (Castellano Mdel et al., 2004). Methyl jasmonate (MeJA) was also reported to affect initiation of DNA replication by inhibiting the pre-replication complex (Noir et al., 2013; **Figure 6**).

The Kip related proteins (KRPs) are direct inhibitors of CDK activity (ICKs). ICK1 inhibits the CDKA/CYCD complex in response to negative stimuli like abscisic acid (ABA; Wang et al., 1998; Van Leene et al., 2011). Kinematic analysis showed that the overexpression of *KRP* genes inhibits cell division rate, resulting in serrated leaves of reduced size with fewer but enlarged cells (De Veylder et al., 2001; Kang et al., 2007). Beemster et al. (2006) could simulate the effect on cell numbers using a computational model. Mutation of a single *KRP* gene does not have any effect on leaf growth, while down-regulation of multiple *KRP* genes increases final leaf area and cell proliferation (Cheng et al., 2013). The mechanism behind this enhanced leaf growth is yet to be explained. Auxin and CK activate CDKA and CYCD, while KRP4 transcription is down-regulated (Cho et al., 2010). It has been reported that auxin signaling is translated into modified *KRP* expression through PRZ1-mediated chromatin remodeling (Anzola et al., 2010). The mutual antagonistic effect of CDKA;1 and KRPs was highlighted in a model of the G1/S transition control in pollen (Zhao et al., 2012). The plant-specific F-box protein F-BOX LIKE 17 (FBL-17) is central in the proposed regulatory network, in particular by mediating the degradation of KRP1,3,4,6,7 (Kim et al., 2008). The plant-specific SIAMESE (SIM)/SIAMESE RELATED (SMR) family also inhibits CDK activity in a number of specific tissues, for instance the repression of the mitotic cycle in trichomes (Walker et al., 2000). GA is proposed to promote mitotic cycles by lowering expression of both KRP and SIM. CKIs act in a DELLA-dependent manner (Achard et al., 2009) and enhance expression of E2Fe/DEL1 (Claeys et al., 2012).

After DNA duplication, cells enter the G2 phase to prepare them for division through mitosis. CDKA and CDKB as well as CYCA, CYCB, and CYCD are involved in this process. The plant-specific B-type CDKs are subdivided into two groups: CDKB1 (with CDKB1;1 and CDKB1;2) and CDKB2 (with CDKB2;1 and CDKB2;2). CDKB1 accumulates from late S to M phase while CDKB2 is specifically expressed from G2 to M phase (Menges et al., 2005). Overexpression of a dominant negative *CDKB1;1* causes early exit from the M phase, which increases the ploidy level in the leaf (Boudolf et al., 2004b). It has been reported that CDKB1;1 forms a functional complex with CYCA2;3 to trigger mitosis in *Arabidopsis* (Boudolf et al., 2009). Inhibition of *CDKB2;1* and* CDKB2;2* via the expression of an amiRNA leads to a dwarf phenotype with an abnormal SAM (Andersen et al., 2008). Jasmonates (JAs) cause G2 arrest in tobacco (*Nicotiana tabacum*) Bright Yellow-2 cell cultures by reducing CYCB1;1 and CDKB (Swiatek et al., 2004). Similarly, inhibition of mitotic phase genes by methyl JA causes G2 arrest in *Arabidopsis* cell cultures (Pauwels et al., 2008).

An additional level of regulation of CDKA/CYCD complex activity involves inactivation through phosphorylation by the WEE1 protein kinase. Overexpression of *WEE1* inhibits plant growth by arresting cell division (De Schutter et al., 2007). However, in plants WEE1 is probably not a core cell cycle regulator, but rather a DNA damage checkpoint kinase (Dissmeyer et al., 2009).

Dissmeyer et al. (2009) have proposed and implemented alternative G2 phase modules in the form of mathematical models starting from an existing generic model. The primitive unicellular algae, *Ostreococcus tauri*, contain a bona fide CDC25, which antagonizes WEE1 phosphorylation (Inze and De Veylder, 2006). In *Arabidopsis* a small CDC25 like phosphatase can counteract the role of WEE1 kinase *in vitro* (Landrieu et al., 2004), but this CDC25-like protein has arsenate reductase activity and is most likely not involved in cell cycle regulation (Dissmeyer et al., 2010). The *Arabidopsis* genome therefore lacks a functional copy of the CDC25 gene, which means that generic cell cycle models (Novak and Tyson, 1993) need to be adapted to reflect the situation in plants.

Similar to the G1 phase, the CDK/CYC complex can be activated in G2 by a CDK-activating kinase pathway, involving CDKF and CDKD coupled with CYCH. The activated CDK/CYC complex promotes MYB repeat (MYB3R) transcription factors to bind with M phase Specific Activators (MAS) elements at the promoter region of the target genes. Afterward, MYB3R phosphorylation activates the expression of M phase specific genes such as *KNOLLE*,* CDC20*,* CYCA*, and *CYCB* and *NACK1* (Berckmans and De Veylder, 2009; **Figure 6**).

There are two E3 ubiquitin ligase complexes involved in cell cycle control, SCF-PROTEIN and APC/C, which mark targets for degradation by the 26S proteasome. In analogy to the SCF complex playing an important role in G1 to S phase by degrading cell cycle inhibitors (KRPs; Fulop et al., 2005; Hershko, 2005; Ren et al., 2008) the APC/C is essential for the G2 to M transition. The exit from the M phase is regulated by the degradation of cyclins through ubiquitination by the APC/C in association with the activators CELL CYCLE SWITCH 52 (CCS52) and CDC20 (Fulop et al., 2005; Sullivan and Morgan, 2007; Marrocco et al., 2010).

Overexpression of *APC10* promotes the cell division rate by degradation of CYCB1;1 which causes enlarged leaves (Eloy et al., 2011). Mutation of *HOBBIT*, a CDC27 subunit of the APC, in the SAM leads to accumulation of high levels of the auxin response inhibitor AXR3/IAA17, indicating that its activity would be involved in targeting AUX/IAA proteins for degradation (Blilou et al., 2002). *CCS52* is an important regulator for controlling exit of mitosis. There are two classes of *CCS52* in *A. thaliana*, *CCS52A* (*CCS52A1* and *CCS52A2*) and *CCS52B* (Fulop et al., 2005). A-type CCS52 activators are typically expressed from late M to late S-G2 and regulate the onset of endoreduplication in leaves (Fulop et al., 2005; Lammens et al., 2008). CCS52B, like the APC/C activator CDC20, peaks from early G2 to M phase exit (Fulop et al., 2005; Kevei et al., 2011). Induced expression of *CCS52B* affects branching in trichomes where *CCS52B^OE^* line forms four to five branches, while the wild type has only three branches (Engler et al., 2012). However, more study is needed in particular to understand role of *CCS52B* in leaf development. It has been suggested that complementary phase-dependent expression of A and B-type CCS52 activators enable a fine-tuned APC/C regulation during the cell cycle (Tarayre et al., 2004). Expression of the negative regulator of CCS52A1 activity, ULTRAVIOLET-B-INSENSITIVE 4 (UVI4) peaks at the G1-to-S transition (Heyman et al., 2011) and determines cell number and size of leaves, likely through stabilization of CYCA2;3 required for mitotic cell divisions (Imai et al., 2006; Boudolf et al., 2009; Heyman et al., 2011). Its homolog UVI-Like/OMISSION OF SECOND DIVISION 1 (OSD1) influences meiosis and does not directly impact leaf size. However, it is expressed during the mitotic cell cycle peaking at the G2-to-M transition, possibly preventing endomitosis (=incomplete mitosis; Heyman and De Veylder, 2012). Besides the mechanism of the inhibitory action of the UVI4 and OSD1 regulators, not much is known about the specific targets of the APC/C (Heyman and De Veylder, 2012). Apart from a recent study pointing to CK up-regulating CCS52A1 in the *Arabidopsis* root (Takahashi et al., 2013), not much is known about the role of hormone signaling on APC/C regulation. However, in stress-conditions GA likely modulates APC/C activity through DELLA dependent down-regulation of the UVI4 and DEL1 negative regulators (Claeys et al., 2012).

Despite of the high level of conservation of the core cell cycle machinery (Harashima et al., 2013), many plant-specific features exist. Plants are characterized for instance by a remarkably broad cyclin family with many species-specific isoforms (at least 49 in *Arabidopsis*, *cf.* Sablowski and Dornelas, 2014). The involvement of different orthologous of many core cell cycle genes goes together with a functional diversity which manifests itself in gene expression differences between species, developmental and environmental conditions. Indeed, Beemster et al. (2005) showed by means of a microarray study that the expression profile of roughly half of all cell cycle genes differed between roots and leaf primordia. de Almeida Engler et al. (2009) found generally high expression of core cell cycle genes in leaf primordia, in the lamina of young leaves and in vascular tissue of expanding leaves. A number of cases of developmental stage-specific expression are described in the relevant sections throughout this review. However, for many others the functional significance has yet to be clarified.

### ENDOREDUPLICATION

Generally cells go through a regular cell cycle with the S phase (DNA duplication) followed by the M phase (mitosis). Endoreduplication, endoreplication, endoploidization or, in short, the endocycle is the process whereby DNA replicates repeatedly without alternating divisions through mitosis, causing a high ploidy level in the cell.

**FIGURE 7:**
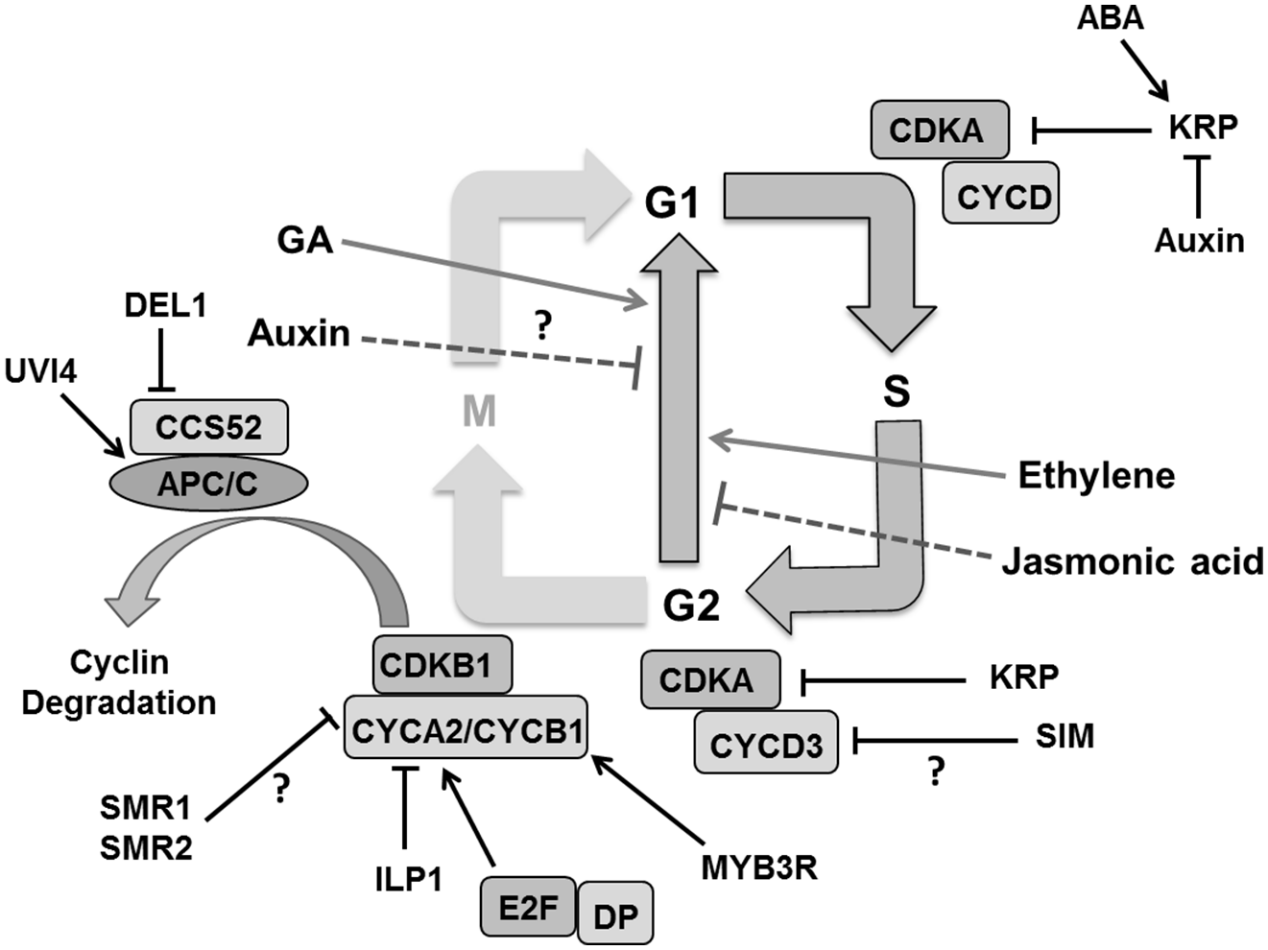
**Regulation of endoreduplication.** CDKA/B and CYC activity is inhibited by cell cycle inhibitors KRPs/SIM/SMRs that induce endoploidization. Cyclin A is inhibited by the ILP1 proteins whereas down-regulation of MYB3R causes decreased CYCB and ultimately induces endoploidization. Proteolytic degradation of G2–M cyclins by the APC/C complex causes endocycle onset. Other factors like, UVI4 and DEL1 suppress the endocycle by inhibiting the APC/C complex. Plant hormones like auxin and jasmonic acid suppress the endocycle whereas gibberellins (GA), abscisic acid (ABA), and ethylene stimulate it by regulating expression and activity of different components (pointed and T shaped arrows indicate positive and negative regulation and question mark shows the unknown regulation, respectively).

To establish endoreduplication, the CDK activity essentially has to be kept low enough and several ways have been proposed to achieve this (Berckmans and De Veylder, 2009; De Veylder et al., 2011). For instance, *CYCD3;1* which is specifically expressed in proliferating tissues, reduces endoploidization (Dewitte et al., 2003). CDKB1 activity is also essential for the G2 to M transition (Beemster et al., 2005). Overexpression of a dominant negative *CDKB1;1* interferes with cell cycle progression causing G2 arrest (Boudolf et al., 2004a). CDKB1;1 forms an active complex with CYCA2;3 to suppress endoreplication in the leaf (Boudolf et al., 2009). Loss of *CYCA2;3* function increases ploidy in mature leaves (Imai et al., 2006). The *INCREASE LEVELS OF PLOIDY* (*ILP1*) gene, which encodes a protein homologous to the C terminal region of mammalian GC binding factor, is proposed to be involved in transcriptional repression of A2-type cyclins (Yoshizumi et al., 2006). Expression of B-type cyclins, on the other hand, was repressed by decreased phosphorylation of three-repeat MYB proteins (MYB3Rs; Ito et al., 2001; De Veylder et al., 2011). The E3 ubiquitin ligase complex, APC/C coupled with CCS52 influences endocycle onset by controlling proteolytic degradation of G2-M specific cyclins like CYCB1;1 and CYCB1;2 (Kasili et al., 2010) as well as CYCA2;3 (Boudolf et al., 2009; **Figure 7**). CCS52A1 and CCS52A2 knockout plants have reduced DNA ploidy levels in leaves (Lammens et al., 2008; Kasili et al., 2010). The previously mentioned plant-specific CCS52A1 inhibitor UVI4 is likely involved in securing the G2-to-M transition and therefore preventing endocycle onset (Heyman and De Veylder, 2012). Cells with increased ploidy levels in *osd1* cotyledons and the developmentally severely compromised *uvi4 osd1* suggest some functional redundancy between UVI4 and its homolog OSD1 (Iwata et al., 2011; Cromer et al., 2012). Mutation in SAMBA, a plant specific subunit of the APC complex which probably activates A2-type cyclin degradation, induces enhanced endoreplication in *Arabidopsi*s leaves (Eloy et al., 2012). Other factors such as the DP/E2F like transcription factor E2Fe/DEL1 are involved in controlling APC/C activity. Down-regulation of DEL1 triggers the expression of the *CCS52A2* gene, forcing cells to enter endoreduplication (Lammens et al., 2008).

The plant specific cell cycle inhibitor *SIM* gene that encodes for a member of the *SMR* family also plays a role in endoreduplication (Walker et al., 2000; Churchman et al., 2006). A mutation in *SIM* causes repressed endoreplication leading instead to mitotic divisions in leaf trichomes. SIM interacts with CDKA;1 and D type CYCs (Churchman et al., 2006), and it was suggested that inhibition of CDKA:CYCD3 complexes might be the mechanism responsible for its role in endoreduplication onset (De Veylder et al., 2011). Indeed, CYCD3;1 overexpression inhibits endoreduplication in *Arabidopsis* leaves (Dewitte et al., 2003), whereas a *cycd3* triple mutant displays premature onset of endoreduplication in young leaves (Dewitte et al., 2007). Other SIM family members, such as SMR1/LGO, might also promote polyploidization (Roeder et al., 2010). In fact, SMR1 and SMR2 interact with CDKB1;1 and its interactor CYCB2;4 associates with SMR11 (Van Leene et al., 2010).

Contrary to the emerging insights into endoreduplication onset, it is only poorly understood how the endocycle is sustained. It has been envisaged that the cell cycle inhibitor *KRP* controls CDKA activity by inhibiting the CDKA/CYCD complex to maintain the CDK oscillations needed for DNA replication in the endocycle. KRPs were reported to regulate mitosis and endoreplication in a dose dependent manner where low concentrations promote the endocycle while high levels cause cell cycle arrest (Verkest et al., 2005b). On the one hand, overexpression of *KRP2/KRP5* in mitotically active cells inhibits cell division and enhances endoreplication (Verkest et al., 2005a; Jegu et al., 2013) while on the other hand its overexpression in postmitotic cells inhibits endocycle in *Arabidopsis* leaves (Schnittger et al., 2003). This implicates that *KRPs* are an important candidate for the regulation of rate and duration of endoreduplication in expanding leaf cells.

*Arabidopsis* leaves, cells enter into the endoreduplication process as a consequence of decreasing auxin concentrations. It has been observed that the mutants in auxin signaling, biosynthesis and transport show a rapid transition from mitotis to endocycle causing increased ploidy level in cotyledons (Ishida et al., 2010) but the detail of this mechanism is still not known. Ethylene and GAs are hypothesized to positively affect endoreduplication (Gendreau et al., 1999; Perazza et al., 1999; Swain et al., 2002). JAs were shown to inhibit cell proliferation as well as endoploidization in a *COI1* (encoding an F-box protein which is a part of the SCF complex) dependent manner in *Arabidopsis* leaves (Noir et al., 2013). It also negatively regulates the expression of key determinants of DNA replication like CDC6A (Noir et al., 2013).

Despite of our increasing knowledge on the molecular mechanism of endoreduplication, its actual function remains ambiguous with proposed roles in promoting cell expansion, stress resistance or DNA damage protection (De Veylder et al., 2011). In any case, modulating CDK–CYC activity levels in various ways remains a central principle. This pertains to the ubiquitin dependent degradation of KRPs or the mechanism by which plant specific cell cycle inhibitors (SIM/SMR) as well as developmental and environmental signals influence the endocycle. Roodbarkelari et al. (2010) have modeled endocycle onset in *Arabidopsis* trichomes with KRP and the CULLIN4 ubiquitin ligase controlling G1/S and SIM and APC/C controlling G2/M transitions. Computational approaches to predict tissue distributions of cell ploidy combined with *in vivo* ploidy maps (Boudolf et al., 2004b) would provide powerful insights to better understand its regulation and relationship to cell expansion.

### REGULATION OF TRANSITION BETWEEN CELL DIVISION AND EXPANSION

**FIGURE 8:**
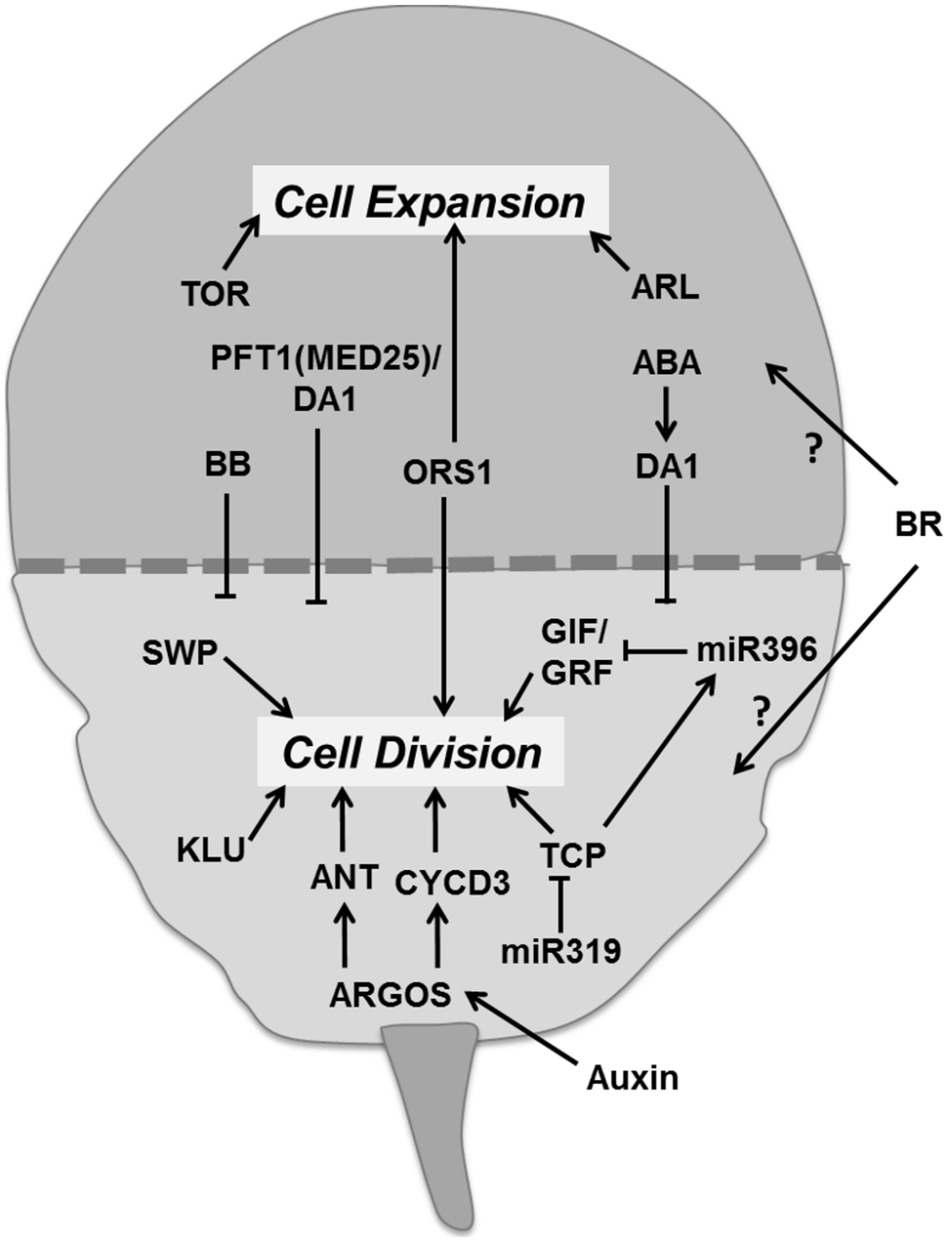
**Regulation of the transition between proliferation and cell expansion.** The transition between division and expansion is shown by a dashed line which separates these two growth processes according to their regulators. ARGOS promotes cell division *via* DNA binding protein ANT and CYCD3 which is regulated by auxin. TCP and GIF/GRF transcription factors promote division and are negatively regulated by miRNAs. Other factors like KLU and SWP also promote proliferation. Some factors like ORS1 have a positive influence on division as well as expansion. Cell expansion is directly controlled by the TOR pathway and ARL. Whereas, other regulators like BB, MED25, and DA1 control the timing of proliferation. Abscisic acid promotes transition at least in part by regulating DA1 whereas brassinosteroids with unknown molecular mechanism (pointed and T shaped arrows indicate positive and negative regulation of the particular process and question marks show unknown mechanisms, respectively).

Leaf development involves two major phases. The first phase is dominated by proliferative activity and the second phase by cell expansion (**Figure 8**). There is a correlation between cell division activity and organ growth, so the timing of cell division has a large influence on the final leaf size (Korner et al., 1989; Meyerowitz, 1997; Gonzalez et al., 2012). As cell division ceases the cell continues expanding. This transition from division to expansion is manifested as a cell cycle arrest front which remains fixed at some position for a particular time period and then moves rapidly toward the base of the leaf blade (Andriankaja et al., 2012). Several regulators appear to control the transition from proliferation to expansion. Auxin plays an important role in the transition phase. It induces the expression of *AUXIN-REGULATED GENE INVOLVED IN ORGAN SIZE* (*ARGOS*) gene, encoding for an ER localized protein of unknown function (Hu et al., 2003). Overexpression and down-regulation of *ARGOS* increases and decreases leaf size, respectively. It regulates the action of a DNA-binding protein ANT (AINTEGUMENTA) and of CYCD3;1 (Hu et al., 2003; **Figure 8**). Loss of function of *ANT* blocks the increase in leaf growth in *ARGOS* overexpressing plants. The *Arabidopsis* ORGAN SIZE RELATED1 (ORS1) shares a conserved domain with ARGOS and ARGOS LIKE (ARL), and positively regulates cell division and expansion in the leaf (Hu et al., 2003; Feng et al., 2011). Like the ANT family proteins, the GROWTH REGULATING FACTOR (GRF) and TCP transcription factors are essential regulators of leaf growth (**Figure 8**). The *Arabidopsis* GRF family comprises nine members. Overexpression of *AtGRF1* and *AtGRF2* results in larger leaves whereas the *grf1/2/3* triple mutant reduces leaf size, both as a result of alterations in cell size (Kim et al., 2003). Overexpression of *GRF5* increased cell number with prolonged growth, whereas the *grf5-1* mutant shows narrow leaves with reduced cell numbers. GIF1 (GRF-INTERACTING FACTOR1), also known as ANGUSTIFOLIA3 (AN3) interacts with GRF5 (Horiguchi et al., 2005). *GIF1* overexpression increases leaf size with leaves having more cells, whereas its absence reduces cell proliferation (Lee et al., 2009).

miR396 negatively regulates six members of *Arabidopsis GRF* together with *GIF1* (Liu et al., 2009; **Figure 8**). Interestingly, overexpression of miR396 in a mutant deficient for *GRF1* reduces SAM size (Rodriguez et al., 2010). The miR396 targeted GRFs are also essential for leaf polarity (Wang et al., 2011). The TCP family of transcription factors regulates the expression of miR396 and miR319 (Palatnik et al., 2003; Rodriguez et al., 2010). A point mutation in the miR319 target site of *TCP4* induces miR396 which in turn decreases *GRF* expression and results in smaller leaves (**Figure 8**). Similarly, overexpression of *TPC4* decreases leaf size (Rodriguez et al., 2010). Transcription factors from the TCP family such as CINCINNATA (CIN) of *Antirrhinum*, LANCEOLATE (LA) of tomato and CIN-TCPs of *A. thaliana* control cell cycle arrest (Nath et al., 2003; Palatnik et al., 2003; Ori et al., 2007). In *Arabidopsis*, up-regulation of miR319 in the *jaw-D* mutant reduces the expression of *TCP2*,* TCP3*,* TCP4*,* TCP10*, and* TCP24* producing large and wrinkled leaves (Palatnik et al., 2003). Down-regulation of single, double, and triple *TPC* genes resulted in proportional increase in leaf size and crinkliness (Schommer et al., 2008).

In addition to these transcription factors, other genes are also essential to promote the transition from division to expansion. The putative ubiquitin binding protein DA1 and the E3 ubiquitin ligase BIG BROTHER (BB) also known as ENHANCER OF DA1-1 (EOD1) controls organ size by restricting the duration of cell proliferation (**Figure 8**). In the *da1-1* mutant, the production of a dominant negative protein negatively affects both DA1 and the DA1-related (DAR) protein and the overexpression of DA1 results in large leaves with increased cell numbers. ABA induces the expression of DA1, whereas the *da1-1* mutant was less sensitive to ABA, implicating a role for ABA in determining final leaf size through control of mitotic exit (Li et al., 2008). Mediator complex subunit 25 (MED25 also known as PFT1), functions together with DA1 in controlling leaf growth by restricting cell proliferation (**Figure 8**). Overexpression of MED25 causes smaller leaves with reduced cell numbers and cell sizes, whereas a loss of function mutant enhances organ size with increased duration of cell proliferation and expansion (Xu and Li, 2011). Loss of function mutation of the RING-finger protein encoding BB leads to enlarged leaves and small changes in expression levels substantially alter organ size suggesting it controls cell division and leaf size in a dose dependent manner (Disch et al., 2006). The *KLUH* (*KLU*)*/CYP78A5* gene, encoding for a cytochrome P450, required for generating a mobile growth signal distinct from the classical phytohormones, is also an essential regulator for leaf size control. Overexpression of *KLU* induces enlarged leaves having more cells whereas in the *klu* mutant premature arrest of cell proliferation causes smaller leaves (Anastasiou et al., 2007; Stransfeld et al., 2010). The *SWP* gene encodes a protein with similarities to subunits of the Mediator transcriptional regulatory complex of RNA polymerase II. It also plays a role in defining the period of cell proliferation. In the *swp* mutant leaf size was reduced due to less cells, which was partially compensated by an increase in final cell size (Autran et al., 2002; **Figure 8**).

Like auxin, sugar signaling controls leaf growth possibly via the ARGOS pathway (Hu et al., 2003; Wang and Ruan, 2013). BR also regulates leaf growth by controlling cell division and expansion. The BR deficient mutant *constitutive photomorphogenesis and dwarfism* (*cpd*) produces smaller leaves with fewer cells of reduced size (Zhiponova et al., 2013); however, the molecular mechanism controlling this process is yet to be clarified. The progressive general cell proliferation arrest front of epidermal and mesophyll cells is followed by a second cell cycle arrest front for dispersed meristematic cells (DMCs) that is controlled by the putative transcription factors PPD1 (PEAPOD1) and PPD2 (White, 2006).

The transition to the expansion phase is essentially dependent on the regulators of cell cycle arrest. Many factors have been implicated in the regulation of the cell division arrest front. An important question is how the spatiotemporal dynamics of the arrest front could be explained. Coordination through one or more gradients of (non-cell autonomous) growth regulators appears to be the most likely mechanism. However, Efroni et al. (2008) hypothesized a mechanism for organ differentiation through an internal self-advancing sequential maturation program, where rate and time of advancement is regulated by a cell-autonomous developmental clock (timed) program. CIN-TCPs would play a governing role in this mechanism for the leaf. Sufficient details for building simulation models of the transition phase still appear to be lacking in *Arabidopsis*. However, in the monocotyledonous maize leaf a clearer picture is arising, where a peak in the activity of GA is instrumental in regulating the spatial location of the transition (Nelissen et al., 2012). Possibly models that will be developed for this monocotyledonous system can be adapted to better understand the same process in the *Arabidopsis* leaf.

### TURGOR DRIVEN CELL GROWTH

**FIGURE 9:**
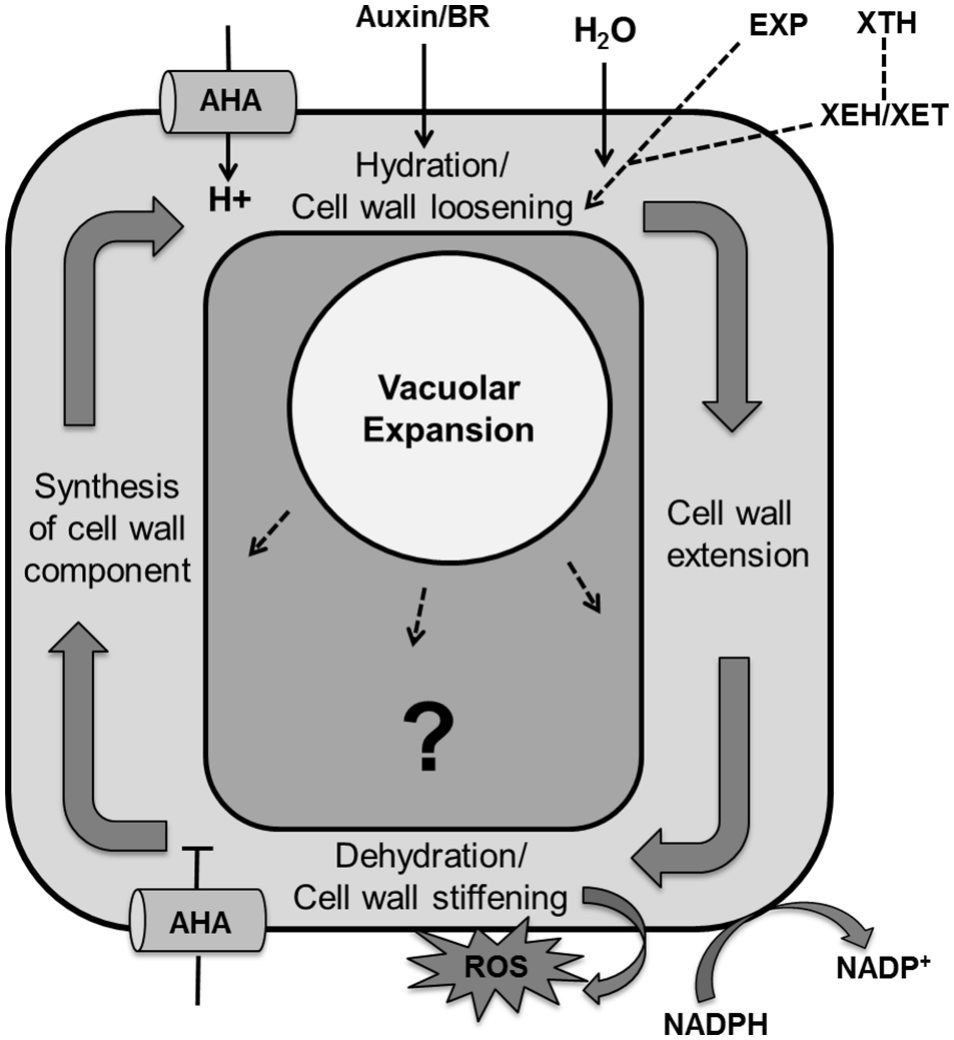
**Regulation of the cell expansion process with unknown molecular mechanism.** Cell expansion is the result of vacuolar enlargement as well turgor driven cell wall yielding. The vacuole expands while taking up water and solutes whereas turgor driven cell wall yielding is the result of multiple steps, including hydration and cell wall loosening, cell wall extension by turgor pressure, dehydration/cell wall stiffening by release of apoplastic reactive oxygen species, cross-linking and dehydration and lastly synthesis and accumulation of cell wall components. Cell wall loosening is controlled by expansin (EXP) proteins and the xyloglucan endohydrolase (XEH) and xyloglucan endotransglucosylase (XET) activities of xyloglucan endotransglucosylase/hydrolases (XTHs). Auxin and brassinosteroid (BR) enhance activity of P-type plasma membrane proton ATPase (AHA; pointed and T shaped arrows indicate positive and negative regulation and question mark shows the unknown regulation, respectively).

Cell expansion is an essential step in determining final leaf size that is governed by different mechanisms in each stage of cellular development. Expansion in meristematic cells is determined by both increases in cytoplasmic and nuclear volume whereas in differentiated tissues it is mainly determined by turgor-driven vacuolar enlargement that allows the accumulation of water and solutes (Wolf et al., 2012; Sablowski and Dornelas, 2014). Turgor driven cell expansion is the result of multiple steps like cell wall relaxation to accommodate water uptake, wall extension by turgor pressure, dehydration/cell wall stiffening and the accumulation of cell wall components (Cosgrove, 2005; Wolf et al., 2012; **Figure 9**).

In the plant cell wall cellulose microfibrils are associated through hemicellulose tethers to form the cellulose–hemicellulose network, which is embedded in a pectin matrix. Primarily, auxin or brassinolide induce activity of P-type plasma membrane proton ATPase (AHA) that causes acidification of the apoplast and in turn activates hydration and cell wall loosening by EXP proteins and xyloglucanendotransglucosylase/hydrolases (XTHs), xyloglucan endohydrolase (XEH), and xyloglucan endotransglucosylase (XET; Yokoyama and Nishitani, 2001; Rose et al., 2002; Caesar et al., 2011; Wolf et al., 2012). Dyson et al. (2012) developed a model of hemicellulose dynamics in an expanding cell wall, showing how the action of XTH and EXP family enzymes determine yield and extensibility of the wall as encapsulated by the classical Lockhart equation (Lockhart, 1965). The mechanism controlling cell wall swelling/hydration is yet to be clarified. Antisense and sense constructs of the *Arabidopsis EXP10* gene produce smaller leaves with altered morphology and larger leaves having bigger cells, respectively (Cho and Cosgrove, 2000). Wall hydration allows cell wall extension through structural alterations. Cell wall relaxation stretches the plasma membrane which promotes opening of Ca^2+^ channels. The resulting increase in cytoplasmic calcium affects growth by inhibiting P-ATPases that cause alkalization of the apoplast and inhibition of EXP activity. It also activates NADPH-oxidase which promotes secretion of superoxide into the cell wall, which is further converted into hydrogen peroxide. These reactive oxygen species promote cross linking of cell wall components, which causes cell wall dehydration and strengthening. At the end, wall thickness is reinstated by biosynthesis of membrane lipids, cell wall components and proteins, and appropriate channelization of these materials to their final cellular destination (Wolf et al., 2012).

Auxin does not always promote cell expansion as its concentration has also been observed to fall during leaf expansion (Braun et al., 2008), suggesting a more complicated dose–response relation. The *yucca* and *sur* mutant of *Arabidopsis* have elevated auxin levels and smaller leaves (Boerjan et al., 1995; Zhao et al., 2001). Mutation in *EXIGUA* (*EXI*) genes, which encode for different subunits of cellulose synthase complex required for secondary cell wall biosynthesis, produces small leaves having defects in cell expansion (Rubio-Díaz et al., 2012). Growth anisotropy, the existence of directions with distinct growth properties is determined by the orientation of the stiff cellulose microfibrils, which in turn is controlled by the orientation of cortical microtubule (CMT) arrays guiding cellulose synthase (Paredez et al., 2006). Uyttewaal et al. (2012) showed by experimental and modeling approaches that the microtubule severing protein katanin mediates the response of cells to mechanical stress in the *Arabidopsis* SAM. The alignment between PIN1 polarity and microtubule orientation in the SAM indicates a tight biophysical coupling between morphogenesis and auxin transport as further corroborated by mathematical modeling (Heisler et al., 2010).

Cell ploidy level is strongly correlated with mature cell size in many plant species (Sugimoto-Shirasu and Roberts, 2003). Alteration in genes specific for G2–M transition affects the onset of endocycle with earlier onset typically leading to enhanced ploidy. A notable exception is down-regulation of *Arabidopsis REGULATORY PARTICLE AAA-ATPASE* (*RPT2a*), which encodes a subunit of the 26S proteasome that causes enlarged plant organs having less but bigger cells. DNA content was higher in some organs but not in all which suggests that the increase in organ size was not the result of endoploidization (Kurepa et al., 2009). Another exception is *KRP2* overexpression in *Arabidopsis*, which does not alter timing of cell cycle exit, but induces fewer and enlarged cells in combination with lower endoploidy levels (De Veylder et al., 2001). A recent study of different *Arabidopsis* mutant and transgenic lines with altered cell sizes showed strong differences in the effect of a same doubling of nuclear ploidy levels, by tetraploidization, on mature cell size (Tsukaya, 2013a). This indicates that genetic factors strongly affect and complicate the general relationship between endoploidy and size, by thus far unknown mechanisms.

### CELL DIFFERENTIATION

In the process of leaf development, cells have the ability to differentiate into distinct cell types such as guard cells, vascular tissue cells, and trichomes, enabling them to perform diverse specialized functions. All these cell types develop from undifferentiated proliferating cells in the young primordium under the control of regulatory pathways that are increasingly being elucidated.

#### Guard cell formation

**FIGURE 10:**
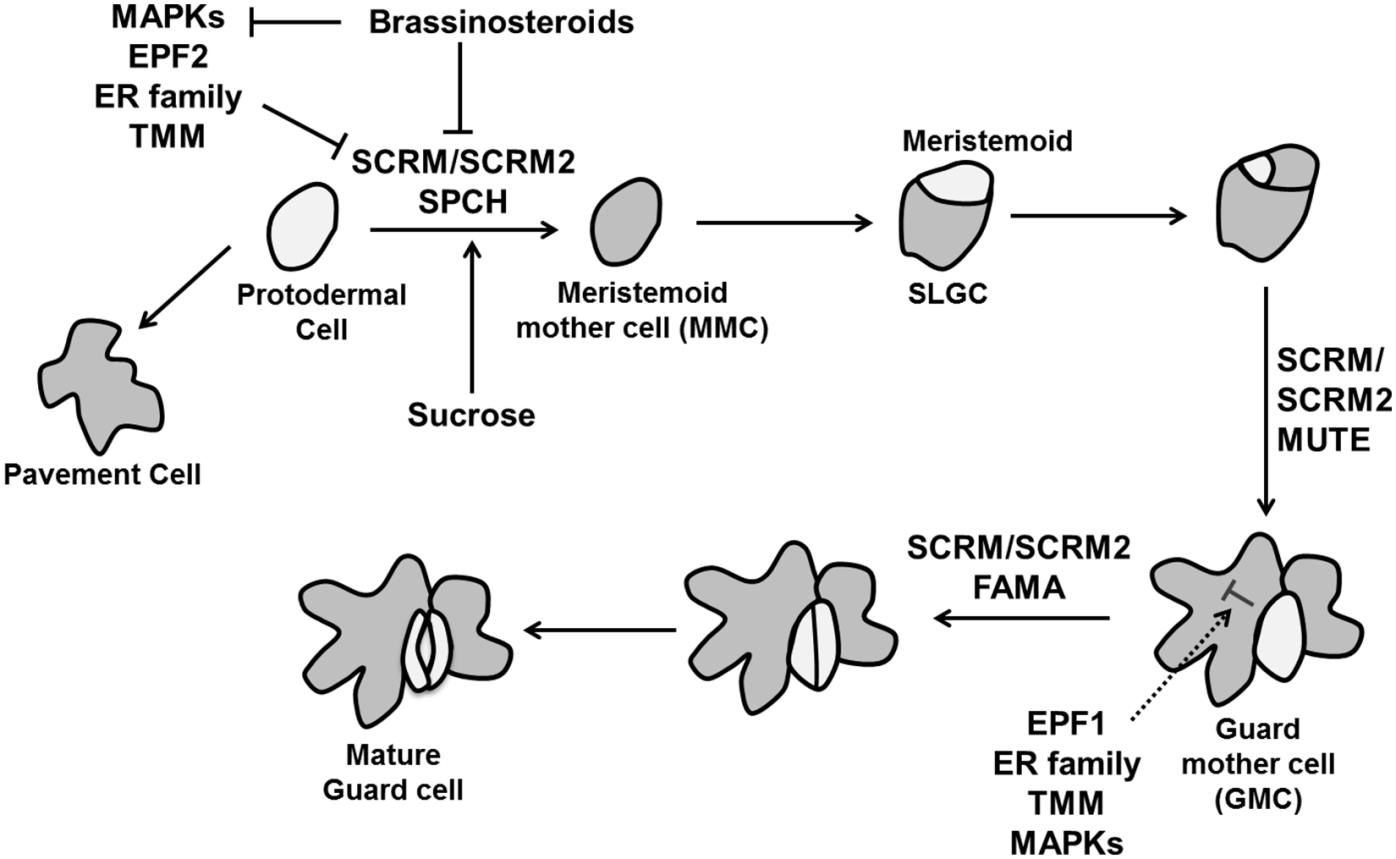
**The control of stomatal development.** Stomatal fate is determined by three transcription factors, SPEECHLESS (SPCH), MUTE, and FAMA. Specification of stomatal lineage where conversion of a protodermal cell into a meristemoid mother cell (MMC) is regulated by SPCH, MUTE controls the transition from meristemoid to guard mother cell (GMC) and FAMA is essential to make functional guard cells from GMC. The MAPK signaling cascade including the MAPK kinase YODA, MPKK4/5/7/9 and MAPKs (MPK3/6), EPIDERMAL PATTERNING FACTORs (EPF1 and EPF2) perceived by TMM and the ER family inhibit stomatal identity in non-stomatal cells. Brassinosteroids negatively regulate SPCH as well MAPKs simultaneously (pointed and T shaped arrows indicate positive and negative regulation, respectively).

In *Arabidopsis*, guard cell development is initiated by an asymmetric cell division of a protodermal cell. The two daughter cells obtain different identities; the larger one maintains protodermal cell identity, whereas the smaller one becomes a meristemoid mother cell (MMC). The MMC divides asymmetrically to produce a larger stomatal lineage ground cell (SLGC) and smaller meristemoid. Subsequently these SLGCs give rise to new meristemoids by asymmetric division. The meristemoid can differentiate into a guard mother cell (GMC), which divides symmetrically to form a pair of guard cell precursors, which further differentiate into guard cells (Vaten and Bergmann, 2012). Two closely related two-MYB-repeat transcription factors, FOUR LIPS (FLP) and MYB88 restrict this final symmetric division to one (Lai et al., 2005). Interestingly, termination of the final division happens through transcriptional repression of the core cell cycle genes CYCA2;3 and CDKB1;1 (Xie et al., 2010; Vanneste et al., 2011). Transition of individual cell into the stomatal lineage is regulated by three helix–loop–helix (bHLH) transcription factors: SPEECHLESS (SPCH), MUTE, and FAMA (Ohashi-Ito and Bergmann, 2006; MacAlister et al., 2007; Pillitteri et al., 2007; **Figure 10**).

The initial asymmetric division when protodermal cells enter the stomatal lineage is controlled by SPCH. Overexpression of *SPCH* initiates extra asymmetric cell divisions while no stomatal lineage was found in the *spch* mutant. *MUTE* is essential to transform a meristemoid into a GMC. Loss of function mutation of *MUTE* leads to the production of stomatal precursors but no stomata, whereas its overexpression converts the whole epidermis into stomata (MacAlister et al., 2007; Pillitteri et al., 2007). Lastly, *FAMA* is required for the conversion of GMCs into guard cells. The GMC divides rapidly in *fama* mutants, but the daughter cells do not differentiate, producing a row of parallel cells (Ohashi-Ito and Bergmann, 2006). A second group of bHLH proteins are INDUCER OF CBF EXPRESSION1/SCREAM (ICE1/SCRM) and SCRM2, which associate with SPCH, MUTE, and FAMA to activate sequential stomatal fate transition (**Figure 10**). Gain of function *scrm-D* causes conversion of epidermal into stomatal cell identity and loss of *SCRM* and *SCRM2* resembles *spch*, *mute*, and *fama* mutant (Kanaoka et al., 2008).

Intracellular signaling pathway analysis revealed that stomatal patterning is regulated by interaction among three leucine-rich repeat receptor kinases (LRR-RLKs): ERECTA (ER), ERECTA-LIKE1 (ERL1), and ERL2 (Shpak et al., 2005), peptides of the EPIDERMAL PATTERNING FACOR-LIKE (EPFL) family (Hara et al., 2009) and the LRR-receptor-like protein, TOO MANY MOUTHS (TMM; Nadeau and Sack, 2002; **Figure 10**). EPF1 and EPF2 expressed in GMC and MMC, respectively (Peterson et al., 2010) control the number of guard and non-guard cells. Loss of function mutants of either *EPF1* or* EPF2* produces more stomata, whereas overexpression inhibits stomatal development (Hara et al., 2009). In contrast, another member of the EPF family* EPFL9/STOMAGEN* is a positive intercellular signaling factor involved in stomatal development (Sugano et al., 2010).

Members of the ER family also work as negative regulators with their down-regulation causing over-proliferation of stomata (Shpak et al., 2005). TMM affects stomatal spacing and density and its loss of function mutant *tmm* forms clusters of stomata in leaves (Nadeau and Sack, 2002). These intracellular signals in turn activate a mitogen-activated protein kinase (MAPK) signaling cascade including the MAPK kinase YODA, MPKK4/5/7/9, and MAPKs (MPK3/6) to inhibit stomatal development in neighboring cells (Bergmann et al., 2004; Lampard et al., 2009; **Figure 10**). MAPK mediated phosphorylation negatively regulates SPCH activity (Lampard et al., 2008), whereas the target in later stage of stomatal development is unknown. A recent study adds to the complexity of this network since the BR pathway phosphorylates YODA (Kim et al., 2012) and SPCH (Gudesblat et al., 2012; **Figure 10**). Thus, it is essential to understand the regulation of MAPK pathway in later stages of the stomatal development and shed light on the complex interaction between YODA and SPCH with BR. It is an interesting question how the stomatal lineage is established and which regulators cause the initiation of *SPCH* expression. In relation to that, a polarity-switching model for individual lineage behavior was able to predict the location of the polarity determinant BREAKING OF ASYMMETRY IN THE STOMATAL LINEAGE (BASL) over multiple divisions leading to stereotypical spatial patterns of stomata lineages (Robinson et al., 2011). Sugar signaling is also involved as an early signal, as sucrose, glucose and fructose all induce ectopic stomatal formation by inducing stomatal lineage markers in non-stomatal lineage cells (Akita et al., 2013).

#### Vascular differentiation

**FIGURE 11:**
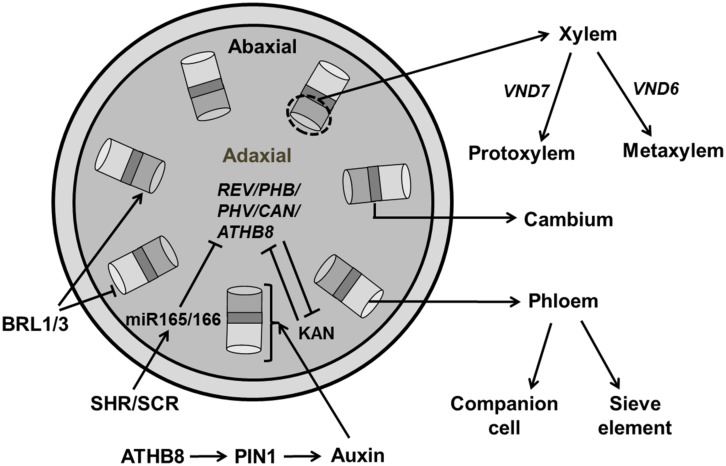
**Regulation of vascular development.** Central regulators for vascular development involves the *REV/PHB/PHV/CAN/ATHB8* genes which are members of HD-ZIP III family and KAN (KANADI). These regulators act antagonistically to maintain xylem and phloem, respectively. Transcription factors, SHR (SHORT ROOT) and SCR (SCARECROW) activate miR165/166, which further inhibits HD-ZIP III. Auxin plays an essential role in regulating vascular formation through PIN1 transporter by early markers like ATHB8. BRASSINOSTEROID INSENSITIVE 1 (BRI1) family, BRI1-LIKE (BRLs) inhibit phloem formation while inducing xylem formation. Expression of *VND6/7* affects the formation of proto/metaxylem (pointed and T shaped arrows indicate positive and negative regulation, respectively).

At the time of leaf initiation, high local concentrations of auxin induce provascular identity leading to the differentiation of midvein and lateral veins preceded by enhanced expression of early markers for vascularization, e.g., ATHB8 (*Arabidopsis* homeobox transcription factor; Scarpella et al., 2004, 2006; Bayer et al., 2009). A Dual Polarization model proposed by Bayer et al. (2009) explains PIN1 protein localization at the time of leaf initiation and midvein formation. Generally, vasculature development begins with the formation of pre-procambium cells, which later differentiate into procambium cells under control of increased auxin flow (Kang and Soh, 2001; Scarpella et al., 2006). Xylem and phloem cells are produced by the vascular meristem with xylem produced on the dorsal (adaxial) side and phloem produced on the ventral (abaxial) side of the procambium. The radial patterning of the vascular bundle is the result of an antagonistic relation between Class III HD-ZIP (Class III Homeodomain Leucine Zipper) in the xylem domain and KAN transcription factors in phloem precursor cells (Jung and Park, 2007; **Figure 11**).

Of all five members of the HD-ZIP III family, *PHV, PHB*, and *REV* are expressed in vasculature, apical, and floral meristems, and the adaxial domain of lateral organs (McConnell et al., 2001; Emery et al., 2003) whereas *ATHB8* and* ATHB15* are exclusively expressed in vascular tissue (Baima et al., 2001; Ohashi-Ito and Fukuda, 2003) and these factors are negatively regulated by microRNA 165/166 (Emery et al., 2003). HD ZIP-III transcription factors are regulated by two members of GARS family of transcription factors, SHR (SHORT ROOT) and SCARECROW (SCR) which activate the genes encoding miR165/166 (Miyashima et al., 2013). Recently, it has been reported that the synchronous expression of *SHR* and *ATHB8* is important for the transition to the pre-procambial cell state that precedes vein formation in leaf (Gardiner et al., 2011). The *phb-6 phv-5 rev-9* loss of function mutant produces abaxialized radial cotyledons in which phloem surrounds xylem (Emery et al., 2003). The quintuple mutant *rev-6 phb-13 phv-11 cna-2 athb8-11/athb8-12* has a severely compromised vascular phenotype similar to the *phb phv rev* triple mutant (Prigge et al., 2005). Loss of *ATHB8* and* ATHB15* has no evident phenotypic effects, though vascular development is slightly perturbed in *athb15*.

The KAN family that belongs to the GARP [Golden2, ARR, and Chlamydomonas regulatory protein of Psr1-type transcription factors], is also essential for vasculature development. The *kan1 kan2 kan3 kan4* quadruple mutant makes abnormal vascular bundles where xylem is surrounded by phloem (Kerstetter et al., 2001; Emery et al., 2003). The transcription factor encoding genes *ALTERED/PHLOEM DEVELOPMENT* (*APL*; which encodes an MYB coiled-coil transcription factor), *VASCULAR-RELATED* NAC*DOMAIN6* (*VND6*), and *VND7* have a direct effect on xylem identity (Bonke et al., 2003; Kubo et al., 2005). Next to the molecular mechanism regulating vascular development that has been extensively investigated (Scarpella et al., 2006), knowledge of the regulation of these processes by spatial signals such as growth hormones in order to explain the establishment of their spatial distribution in simulation models is also emerging. Various mathematical models were constructed to explore the role of auxin in vasculature development (Scarpella et al., 2006; De Vos et al., 2012). However, a model proposed by Cano-Delgado et al. (2004) highlights the role of BRs in vascular patterning in *Arabidopsis*. BRs is perceived by BRASSINOSTEROID INSENSITIVE 1 (BRI1), a membrane localized LRR-RL kinase which increase xylem and reduced phloem differentiation. The loss of function of members of BRI1 family, BRI1-LIKE1 (BRL1) and BRI1-LIKE3 (BRL3) produces a phenotype of reduced xylem and increased phloem (Cano-Delgado et al., 2004). A mathematical model by Ibanes et al. (2009) shows that BR interacts with auxin for spatial regulation of vascular bundles in shoot inflorescence.

#### Trichome development

**FIGURE 12:**
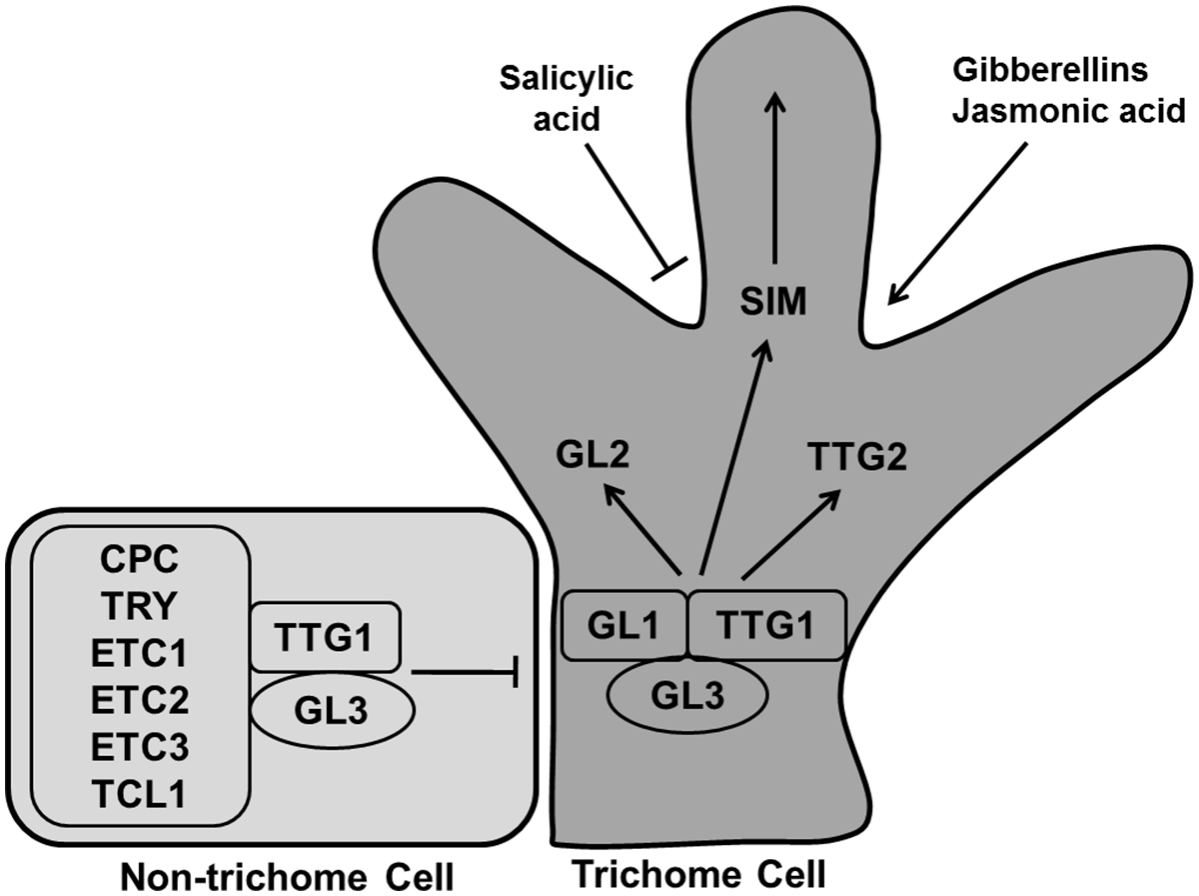
**Regulation of trichome differentiation.** Transcription factors GLABRA1 (GL1), GLABRA3 (GL3), and TRANSPARENT TESTA GLABRA1 (TTG1) forming the MYB/bHLH/WD-repeat complex activates trichome development whereas CAPRICE (CPC), TRIPTYCHON (TRY), ENHANCER OF TRY AND CPCs (ETC1, ETC2, and ETC3), and TRICHOMELESS1 (TCL1) inhibit the process. The MYB/bHLH/WD-repeat complex causes the activation of GL2, TTG2, and SIM to induce trichome differentiation. Trichome production is enhanced by gibberellins and jasmonic acid, while salicylic acid inhibit it (pointed and T shaped arrows indicate positive and negative regulation, respectively).

During leaf development specific epidermal cells convert into leaf hairs or trichomes. Trichomes generally go through three stages for their developmental-cell fate determination, specification and morphogenesis (Hulskamp et al., 1994). Gene products related to trichome formation can be subdivided into positive and negative regulators. The R2R3 MYB transcription factor GLABRA1 (GL1), the bHLH factor GLABRA3 (GL3), and the WD40-repeat factor TRANSPARENT TESTA GLABRA1 (TTG1) are positive regulators for trichome formation (**Figure 12**). The Null mutant *gl1-1* is not fully glabrous, a few trichomes develop at the edges of the late rosette leaf (Oppenheimer et al., 1991; Kirik et al., 2005). The *gl3* mutant shows the same phenotype as *gl1-1* whereas overexpression of *GL3* overcomes the trichome defect of *ttg1* (Zhang et al., 2003). GL1 and TTG1 bind with GL3, forming a MYB/bHLH/WD-repeat complex that activates the expression of its downstream activators GL2 and *TTG2*, causing trichome differentiation (Zhao et al., 2008; Grebe, 2012; Yang and Ye, 2013).

*CAPRICE* (*CPC*), *TRIPTYCHON* (*TRY*),* ENHANCER OF TRY AND CPCs* (*ETC1*, *ETC2*,* and ETC3*), and *TRICHOMELESS1* (*TCL1*) are negative regulators, encoding for R3 MYB proteins (Tominaga et al., 2008; **Figure 12**). A loss of function mutant of *tcl1-1* induces trichome formation and overexpression repressed trichome formation completely in *Arabidopsis* (Wang et al., 2007). The triple mutant *etc2 try cpc* produces trichome at the edges of the leaves. All these small MYB proteins replace GL1 in the MYB/bHLH/WD-repeat complex, rendering it inactive so that the cell remains in the undifferentiated state (Kirik et al., 2004).

Generally, trichome cells go through four endoreduplication cycles for their development, reaching an average DNA content of 32C, whereas other epidermal cells continue to divide (Schnittger and Hulskamp, 2002). It has been observed that the cell cycle related genes like *SIM*, *TRY*, *SlCycB2*, and genes involved in the endoreduplication process also regulate trichome formation. A *sim* mutant was found to have altered ploidy levels affecting trichome development (Schnittger et al., 1999; Walker et al., 2000; Pesch and Hulskamp, 2011). *SIM* is indeed directly targeted by the trichome initiation factors GL1 and GL3 (Morohashi and Grotewold, 2009). Plant hormones also regulate trichome formation with GA and jasmonic acid enhancing trichome number and density while salicylic acid reducing trichome number (Traw and Bergelson, 2003). Still, more study is needed to explore the role of phytohormone signaling pathways in trichome formation.

Two main theoretical models have been proposed to explain trichome patterning in *Arabidopsis* leaves: an activator–inhibitor model and an activator–depletion model. In the activator–inhibitor model, the activator (trimer complex of WD40, bHLH, and MYB factors) triggers its own inhibitor (R3MYB) which moves into the neighboring cell and impedes activation of the complex whereas, the activator–depletion model explains GL3 dependent depletion of TTG1 in non trichome cells (Pesch and Hulskamp, 2009). Computational modeling of the trichome pattern was used by Bouyer et al. (2008) to evaluate these conceptual models indicating that both models may act in concert.

## A SYSTEM’S PERSPECTIVE ON LEAF GROWTH

In Systems Biology the aim is to acquire a mechanistic understanding of biological processes. In most cases the detailed knowledge is formulated in mathematical models that can simulate the behavior of the system and predict the effect of environmental and genetic perturbations. Such predictions can then be experimentally tested and the results used to improve the models further. This way models and experiments reinforce each other leading to increased understanding of the system (Kitano, 2002).

Here we adopted the view that cells are the units that direct development by integrating local signals into developmental decisions. Therefore, to build a mechanistic model for leaf growth we need to be able to model a single cell and its progeny as it progresses from the stem cell niche into the various positions in the mature leaf. The outcome of the integrated behavior of all cells ultimately forming the leaf is an organ of realistic size and morphology. This is currently still a very ambitious goal, but as a first step we addressed here the question what the current state of knowledge is with regards to regulatory networks that operate in cells as they progress in their individual developmental pathway.

During their developmental journey plant cells or rather their cell lineages are exposed to diverse biochemical and biophysical conditions, despite being tied into a symplastic mesh-work. Whereas, their final fate can be very different, ending up as a light harvesting mesophyll cell versus an epidermal hair cell for instance, many similarities exist in the events along their paths starting from the stem cell niche of the SAM. We have associated the different processes described above in separate sections with separate regulatory networks. However, by comparing the corresponding network diagrams, it readily becomes clear that many regulators and relations are shared; indicating that considering them as isolated systems is a radical assumption.

All presented networks are subject to intense investigation and some are far from the finished article, yet from a structural or topological perspective there are some recurring themes or motifs that emerge. A first case is the negative feedback loop which is a typical control structure that works like a thermostat: one factor stimulates a second factor which switches the first one off above a certain threshold. Not surprisingly this motif is active in the SAM where the WUS–CLV interaction ensures that sufficient stem cells are maintained for indeterminate growth, at the same time avoiding over-proliferation. A second case we have encountered is the CDK–APC/C interaction of the cell cycle. Here, the CDK–CYC activity required for cell proliferation eventually turns on the degradation machinery that inactivates CDK–CYC. Rather than providing spatial bounds the latter mechanism confers temporal control on cell proliferation. A somewhat related type of periodicity is a result of the auxin–PIN interaction crucial to phyllotactic patterning but likely also for determining leaf venation and serration (Bilsborough et al., 2011). By polarizing PINs toward auxin maxima, auxin levels are depleted in the surroundings. Another recurring motif is that of mutual negative feedback inhibition which can lead to switch like (bistable) behavior. An example is the proposed role of CDKA–KRP in the G1/S module of the cell cycle (Zhao et al., 2012). Such a motif can also provide a strong basis to support two spatially distinct and stable developmental domains. The antagonistic relation between ARP family and KNOX family transcription factors for instance appears to operate as a mechanism that enables primordium outgrowth while keeping the surrounding regions of the SAM undifferentiated. We have encountered other cases where such a duality appears to be crucial: determination of ab/adaxial leaf polarity on the one hand and vascular differentiation on the other hand are both governed by the antagonistic relation between HD-ZIPIII and KAN family transcription factors. Importantly, here to exert such spatial inhibitory effects additional mobile signals are in principle required, since the before mentioned TFs as far as we know are immobile. Various small RNAs are prime candidates for such a role (Braybrook and Kuhlemeier, 2010). In fact, many superimposed interactions with other factors are typically present to further increase robustness to deleterious perturbations or in contrast to increase the response to important developmental or environmental cues.

As we have seen, how well the described regulatory networks are understood varies considerably. Despite many plant-specific features and intricacies, the universal role and conserved character of the cell cycle has helped in uncovering its regulation to a considerable extent. Nevertheless, the precise functioning of KRPs and ubiquitin mediated degradation in cell cycle transitions remains to be elucidated. For the regulation of the transition between division and expansion for instance the coherency in the corresponding network diagram is weaker indicating that our knowledge is still more scattered and circumstantial. This lack of conceptual understanding is reflected in the absence of published computational models for this process and similarly for others. Our understanding of the regulation of cell growth is also relatively limited, in particular its relation to cell division (Sablowski and Dornelas, 2014). Whereas, the core machinery is relatively well understood, little is known about the way that primary growth determinants such as water or nutrient availability are translated into cell growth differences. The role of the TOR pathway in the regulation of growth and division, crucial in other eukaryotes, is only starting to emerge for plants (Xiong et al., 2013).

Because of the symplastic nature of plant tissue and the lack of a central nervous system, organ growth is more dependent on mobile growth signals that produce local gradients, such as phytohormones, mobile proteins and miRNAs. Since evolution tends to take a parsimonious approach it is not surprising that several growth signals are shared by different processes (and organisms as well). As illustrated above (see **Figure 1**), auxin has indeed been implicated in many developmental stages. If CKs are involved then they typically act antagonistically with auxin and GAs (cf. primordium initiation). BRs at one hand positively regulate many growth processes, while on the other hand they negatively regulate guard cell development. Some of the stress induced hormones such as ABA and ethylene modulate cellular processes for example division, endoreplication and the transition phase. Other growth hormones like JA and salicylic acid exert a negative control on the endocycle and on leaf hair development, respectively. These phytohormones have complex interactions where one affects other’s synthesis, transport, and signaling cascades.

Obviously a generalization of the role of specific hormones would imply over-simplification given the complexity of the regulatory interactions involved. Vernoux et al. (2011) demonstrated indeed that the control of gene expression by auxin not only depends on its distribution but also the expression patterns of the signaling network which consists of over 50 potentially interacting transcriptional activators and repressors. This study further highlights the importance of an integrative strategy which mathematical modeling supported by detailed expression maps, live imaging of biosensors, and high-throughput (interactome) data analysis. Given the crucial and complex role of non-cell-autonomous signals such as phytohormones the development of sensitive (fluorescent) biosensors to monitor their spatial and temporal distribution is an important trend (Brunoud et al., 2012; Shani et al., 2013; Wells et al., 2013).

Other experimental data becoming invaluable for developing improved mathematical models of leaf growth are quantitative growth data ranging from kinematic output (Nelissen et al., 2013) to cellular-resolution digital data extracted form confocal images (Kierzkowski et al., 2012). As repeatedly indicated above, multiple connections exist between the discussed developmental stages, suggesting that an important challenge will be to construct computational models that can reproduce these stages in a spontaneous way. This will likely require a more advanced geometrical representation than a flat plane or a simple sphere or cylinder. Developing coupled dynamical models of different tissues or organs interacting through an interface might provide a useful first step. However, eventually fully integrated three-dimensional models will be developed to grasp the complex cross-talk between various internal and external signals. Next to biological insight, increased computing power, for instance through improved parallelization algorithms, will likely become the limiting factor in that process. Ultimately, a mechanistic model for leaf development should integrate the regulatory networks that control developmental decisions and processes of cells as they migrate in space and time from the SAM to their final position in the leaf. Besides spatially and temporally highly resolved experimental techniques combined with advanced top down data-extraction techniques an important aspect will still remain to apply Ockham’s razor in a sensible way. Choosing a minimal set of variables to produce the desired behavior will present a challenge given the number of factors that are known to be involved or that are still to be discovered. As we have attempted to demonstrate, in a number of cases it is already clear which are the central regulators of the respective regulatory networks and some are indeed central to existing computational models. Furthermore, the non-cell autonomous signals and their gradients will inevitably be part of those future leaf developmental models and connect them to the rest of the plant and even the environment.

## Conflict of Interest Statement

The authors declare that the research was conducted in the absence of any commercial or financial relationships that could be construed as a potential conflict of interest.
